# Dysphagia Prevalence in Progressive Supranuclear Palsy: A Systematic Review and Meta-Analysis

**DOI:** 10.1007/s00455-024-10681-7

**Published:** 2024-03-24

**Authors:** Julia Glinzer, Éadaoin Flynn, Eleni Tampoukari, Isolde Harpur, Margaret Walshe

**Affiliations:** 1 Department of Clinical Speech and Language Studies, Trinity College Dublin, University of Dublin, Dublin 2, Ireland; 2 Department of Voice, Speech and Hearing Disorders, Center for Clinical Neurosciences, University Medical Center Hamburg-Eppendorf, Hamburg, Germany; 3 Department of Speech and Language Therapy, Tallaght University Hospital, Dublin, Ireland; 4 The Library of Trinity College Dublin, Dublin, Ireland

**Keywords:** Dysphagia, Prevalence, Meta-analysis, Aspiration, Progressive supranuclear palsy, Parkinsonian disorders

## Abstract

**Abstract:**

The objective of this systematic review was to determine the prevalence of dysphagia and aspiration in people with progressive supranuclear palsy (PSP). A search of six electronic databases was performed from inception to April 2022. No context restrictions were set. All primary research comprising figures to derive a prevalence rate were included. Two independent reviewers screened search results. Data were extracted by one reviewer. Conflicts were resolved by discussion with a third reviewer. The quality of included studies was assessed using the JBI Checklist for Prevalence Studies. From 877 studies, 12 were eligible for inclusion. Dysphagia had to be confirmed using instrumental assessments, clinical swallowing evaluation, screening, and patient-reported outcome measures (PROM). A random-effects meta-analysis calculated a pooled dysphagia prevalence in 78–89% (95% CI [60.6, 89.1], [78.9, 95.0]). depending on the chosen assessment method, and a pooled aspiration prevalence of 23.5% (95% CI [14.5, 33.7]). The included studies were of moderate quality, with high risk of selection and coverage bias and low to moderate risk of measurement bias. Dysphagia is highly prevalent in a sample of participants with mostly moderately severe PSP. Aspiration occurs in a quarter of this sample and is likely to increase as the disease progresses. Given the low general prevalence of PSP, studies remain at high risk for selection bias. Prospective research should focus on the development of dysphagia in the course of PSP and its subcategories using instrumental assessment and consider all phases of swallowing.

**Registration:**

The protocol of this systematic review was registered on the International Prospective Register of Systematic Reviews (PROSPERO) in April 2021 (registration number: CRD42021245204).

**Supplementary Information:**

The online version contains supplementary material available at 10.1007/s00455-024-10681-7.

## Introduction

Atypical parkinsonian disorders (APD) are characterised by their rapid progression [1] and short survival time in contrast to idiopathic Parkinson’s disease (IPD) [2–5]. Progressive supranuclear palsy (PSP) is the most common type of disorder amongst the APD [6]. The median survival time of PSP from symptom onset ranges from 5.3 to 10.2 years [7]. The pooled prevalence rate of PSP is 7.1 per 100.000 [8] with a mean age of onset of 66.0 (SD = 8.76) years [9].

Dysphagia is the third most common symptom reported in PSP [10]. Dysphagia characteristics of the oral preparatory and oral phase typically presenting in individuals with PSP include impaired bolus control and transport due to impaired tongue control and motility presenting as anterior as well as posterior bolus loss with delayed swallow intitiation and oral residue [11–14]. Reported symptoms of the pharyngeal phase include residue and airway invasion above (penetration) and below (aspiration) the vocal cords [11, 13]. The symptoms in the pharyngeal phase are attributed to reduced speed and extent of movement [15] and reduced pharyngeal pressure [16]. In the esophageal phase, retention and retrograde flow are reported and attributed to sphincter dysfunction and impaired esophageal motility [11, 12, 15–17]. Dysphagia in PSP is reported to develop earlier than in other Parkinsonian disorders [10]. Although, the reported prevalence rates of bulbar impairment vary [18, 19], bulbar impairment manifests itself in the early stages of the disease [9, 19]. In 2–3 years from symptom onset, the initial percentage of people reporting dysphagia is said to increase dramatically with persons reporting impaired swallowing three times more often than impaired speech [9, 20]. Dysphagia affects four out of five people approximately 3 years after symptom onset [9] with an increased need for tube feeding as the disease progresses [9, 18]. Accordingly, dysphagia is a leading symptom in patients admitted to palliative care facilities [21]. Moreover, there exists a significant correlation between latency to dysphagia and total survival time [10]. Earlier dysphagia onset predicts a shorter survival time [7, 22]. Despite acknowledgement of the presence of dysphagia in PSP and its consequences for people, limited data exists on its prevalence.

Lack of data on the prevalence and characteristics of dysphagia in PSP has implications for dysphagia service planning delivery. Given the relatively rapid progression of symptoms, assessment protocols, management options as well as patient education around tube feeding, and palliative care supports need to be carefully planned. To date, there are challenges in understanding the epidemiology of dysphagia in PSP. This relates in part to some methodological limitations regarding dysphagia assessment methods in addition to relatively small sample sizes in existing studies. Most studies comprising larger participant numbers that report PSP disease progression and include the presence of dysphagia base their diagnosis of impaired swallowing on either subjective patient reports, medical chart reviews, or physician opinion.

The diagnostic accuracy of these methods is not strong and this substantially impacts generalisation of the results. These methods of assessment also do not allow for confirmation of dysphagia consequences such as aspiration. Aspiration, defined as airway invasion of food, fluids or saliva below the vocal cords, is considered a contributing factor in the development of aspiration pneumonia [23]. Aspiration pneumonia is stated as the most common cause of death in PSP [2, 24]. However, studies exist that describe dysphagia in PSP based on instrumental assessment methods but to our knowledge, no one has amalgamated evidence from these studies to reach a conclusion regarding the prevalence of dysphagia in PSP.

The objective of this systematic review is to determine the prevalence of dysphagia and aspiration in PSP by adhering to a rigorous methodology and combining single studies according to detailed eligibility criteria concerning the assessment of PSP and dysphagia.

## Methods

This systematic review (SR) and meta-analysis were planned in consideration of the Preferred Reporting Items for Systematic Reviews and Meta-Analyses (PRISMA) Statement [25] and Meta-analysis of Observational Studies in Epidemiology (MOOSE) guideline [26] (see supplementary material). The research protocol was published on the International Prospective Register of Systematic Reviews (PROSPERO) on April 2021 (registration number: CRD42021245204). As this SR focusses on prevalence, the research objective and eligibility criteria were formulated based on the Condition-Context-Population framework [27].

### Eligibility Criteria

Studies were included if they contained data regarding dysphagia prevalence or incidence in participants diagnosed with PSP. Dysphagia diagnosis needed to be based on patient-reported outcome measures (PROM), clinical or instrumental swallowing examination and clearly defined. Dysphagia for the purposes of this study is defined as difficulty in swallowing saliva, food, liquid and comprises of difficulties in the oral, pharyngeal and/or esophageal phase of swallowing. The medical diagnosis of PSP should have been based on neurological investigation. Studies including participants with co-existing conditions, such as nonprogressive neurological or oncological conditions, were only included if data specific to dysphagia caused by PSP could be separated. No age restrictions were set as the mean age of onset is 66 (SD = 8.76) [9]. No restrictions regarding the context (language, geography, or date) were set. All primary quantitative research as well as randomised, and non-randomised controlled trials were included. Data from Grey literature, errata and letters were eligible for inclusion. Secondary research in the form of literature reviews was used as a source to identify further eligible primary research but was excluded from data extraction. Conference abstracts were excluded because they do not allow for a thorough quality assessment. Case studies were excluded as they do not allow for calculation of a prevalence rate.

### Search Strategy

The search strategy (see supplementary material) was developed in consultation with a subject librarian. The following databases were searched from inception to April 2022: PubMed, EMBASE, CINAHL, Web of Science Core, ProQuest Dissertations & Theses, and OpenGrey. Databases were chosen based on thematic relevance and expertise of the subject librarian. Where applicable, controlled vocabulary was combined with free-text terms to avoid missing relevant articles. Free-text terms were consistent across all databases. Controlled vocabulary was adapted for each database because different databased index certain symptoms or syndromes differently. To identify additional sources, citation searching was deployed, i.e., screening bibliographies of relevant papers for additional eligible papers. Hand-searching was not implemented due to time restrictions and the restrictions of the global pandemic preventing access to printed research.

### Study Selection and Data Extraction

Two reviewers independently checked all titles and abstracts of the retrieved results and excluded irrelevant studies. All full texts of the relevant studies were obtained and subsequently independently examined for compliance with eligibility criteria by two reviewers. Agreement of two reviewers was calculated with Cohen’s kappa coefficient (*κ*). COVIDENCE (Veritas Health Innovation) [28] was used to merge search results from the different databases searched, to remove duplicates, to aid with title and abstract screening, as well as full-text review, risk of bias assessment, and data extraction. The extraction form was piloted on two randomly selected studies to assess its suitability and ensure that all necessary data are extracted [29–31]. This extraction form ensures the standardised data extraction across studies. Data were extracted by one reviewer and questions resolved with the research supervisor. Data were extracted regarding study characteristics, eligibility, population, context, and condition. In cases of missing data or unobtainable studies, authors of studies published not later than the last 5 years were contacted. Two weeks after the first unsuccessful contact attempt a follow-up e-mail was sent, if the second attempt was unsuccessful and no feedback was received until completion of data analysis or if contact information could not be obtained, these studies were excluded.

### Quality Assessment

The methodological quality of included studies was assessed with the JBI Critical Appraisal Checklist for Studies Reporting Prevalence Data [32]. It is important to consider the risk of bias of individual studies with regards to the interpretation of results of SR [33].

### Data Synthesis

The characteristics of included studies were synthesised narratively and charted in tabular format. A meta-analysis was planned to calculate the prevalence rates of dysphagia and aspiration. The prevalence rate was defined as the proportion of the number of participants that presented with impaired swallowing or aspiration divided by the number of participants of each study [34]. If data of more than one assessment method was presented, the prevalence figures of the most objective valid measure were used in the analysis to avoid counting participants from one study twice and thus overstating the evidence [35]. Aspiration was defined as material entering the trachea and passing below the vocal folds, with a Penetration–Aspiration Scale [36] score ≥6 or an equal value of another scale. Aspiration needed to be assessed by either fiberoptic endoscopic evaluation of swallowing (FEES) or videofluoroscopic study of swallowing (VFSS) which are both considered to be the reference standards of dysphagia assessment. The meta-analysis of the results was carried out with R Studio (Version 2022.12.0+353) following the instructions of Harrer et al. [37], using the packages meta [38] and metafor [39]. A random-effects model was chosen due to anticipated methodological variability of the assessment of the condition. In this model the variance (*τ*^2^) was calculated with the maximum likelihood estimator. The proportions were transformed with the logit transformation. Due to anticipated small sample sizes, individual confidence intervals were estimated with the Clopper–Pearson method [40]. The proportions were pooled with a generalized linear mixed-effects model as recommended [41]. The results of the meta-analysis were presented quantitatively with 95% confidence intervals (CI) and graphically in a forest plot. Causes of heterogeneity among study results were explored by subgroup analyses if applicable. Heterogeneity of included studies was assessed with the *I*^2^ statistic and interpreted according to different levels of heterogeneity specified by Deeks et al. [42]. Normally a *p* > 0.05 confirms the null hypothesis of no heterogeneity [43]. However, with small sample sizes and a low number of included studies, the strength of *χ*2 is reduced which is why a *p*-value of 0.1 might be more useful [42].

## Results

### Study Selection

**Fig. 1:**
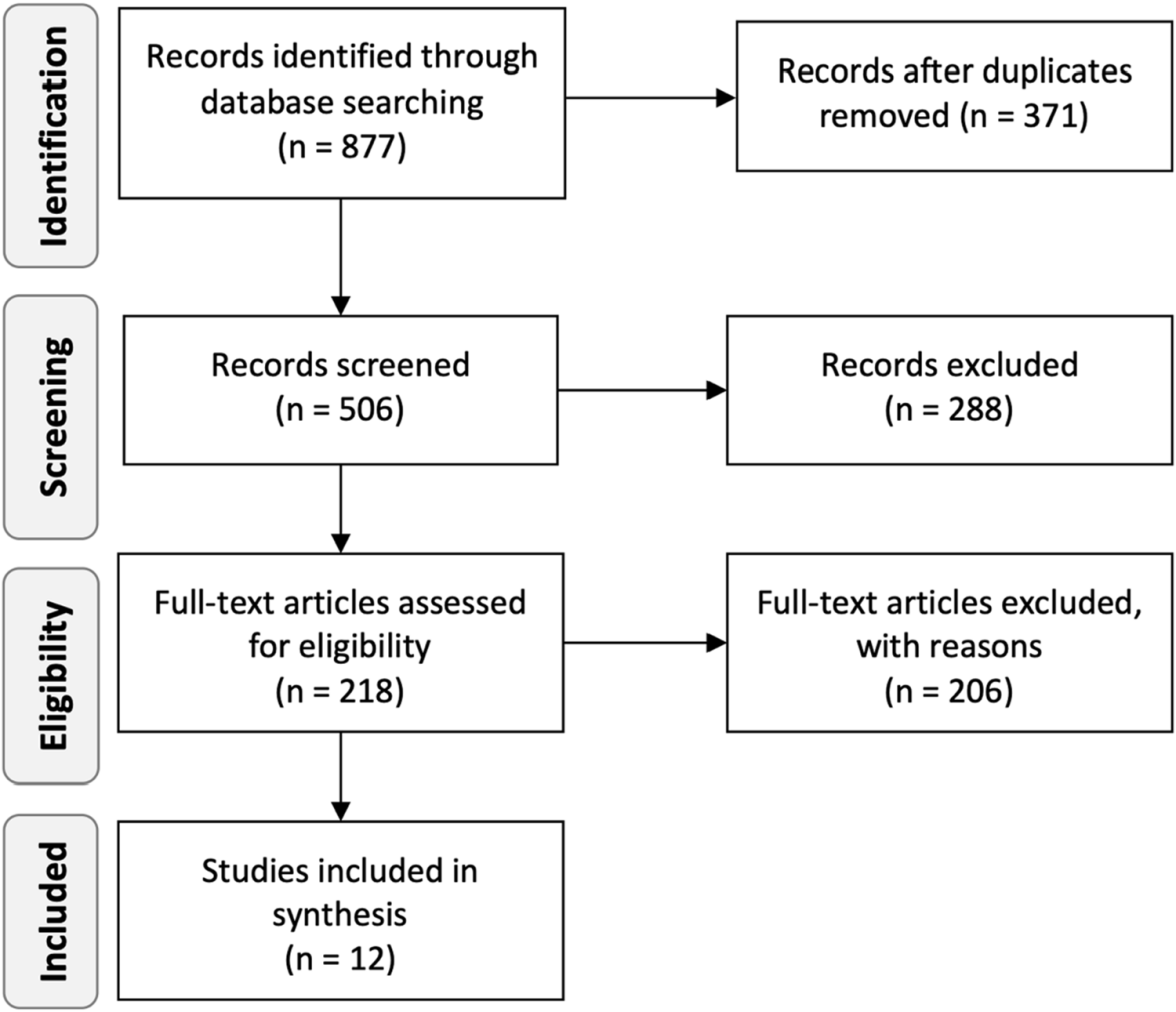
Flow diagram of the review process according to PRISMA

Eight hundred and seventy-seven studies were identified and imported into Covidence (Fig. 1). After removing 371 duplicates and 506 studies based on their titles and abstracts, 218 full texts were assessed for inclusion. The inter-rater agreement during title and abstract screening was 86% (Cohens’s *κ* = 71.3) and during full-text screening was 96.7%, (Cohens’s *κ* = 83.3), which corresponds to good and very good agreement, respectively [44]. Consensus was reached by discussion amongst authors. Persisting disagreements were resolved by a third reviewer. EndNote [45] was used to retrieve full texts. Studies were immediately excluded if neither abstract nor full-text could be obtained, and if they did not include information about the condition and population in the title. Twelve studies of the 218 full-text articles assessed were determined eligible for inclusion. Reasons for further exclusion despite initially meeting the inclusion criteria were e.g., a potential overlap of participants in other publications [11, 46], only including participants with dysphagia [17, 47, 48] or case descriptions [14, 49] (see supplementary material).

### Study Characteristics

#### Participants

The characteristics of included studies are presented in Table 1. The studies were published between 1997 and 2022. All studies were published in English. The median sample size was 24 and ranged from 7 to 491. The age ranged from 44 to 88 years, with a pooled mean age of 70.53 years. The overall male–female ratio was 1.2 (m:f = 492:414). The disease duration ranged from 0 to 13 years. Overall, the severity of PSP was rated as moderate by means of different rating scales.

**Table 1:** Characteristics of included studies regarding study design, population, context, condition, and quality ratings

Authors/year/country of origin/setting	Design	*n*	Age in years (M ± SD)	Sex (m:f)	Disease duration in years (M ± SD)	Disease severity (M ± SD)	Assessment method	Consistency	Prevalence (Dysphagia; Aspiration)	Rating Scale	Dysphagia Severity	Quality JBI Checklist
Cosentino et al. (2020) Italy University and Clinic	Analytic observational, case–control	10	70 ± 6.2	2:9	3.8 ± 2.0	56.2 ± 6.5^a^	FEES, EMG	Liquid, puree, solids	9/10	DOSS (1–7)	4.3 ± 0.5 Excluded: severe dysphagia (DOSS 1–3)	8/16
Clark et al. (2020) USA Clinic	Descriptive observational, cross-sectional	51	71 (MD) (IQR 65–75) [54–86]	26:25	4 (MD) (IQR 2–5) [1–10]	42 (IQR 35–50), [17–67]^a^	VFSS	Liquid, nectar, pudding, solids	45/51 (MBSImP: oral Residue ≥ 2); 8/51 (PAS ≥ 6)	MBSImP PAS FOIS	8/17 Oral Total Score (MD) 2/31 pharyngeal total score (MD); Liquid: 2 (MD), Nectar: 1 (MD), Puree/Solid: 1	12/16
Enver et al. (2020) USA University	Descriptive observational, cross-sectional	23	72.39 ± 6.96 [54–85]	16:7	5.03 ± 2.26 [1.88–10.88]	Not specified	FEES	Liquid	21/23 (PAS ≥ 3); 9/23 (PAS ≥ 6)	PAS	5 (MD), (IQR 3–8), 8 Mode	9/16
Choi et al. (2021) Korea University	Analytic observational, cohort	123	68 ± 6.3	68:55	3.7 ± 2.6	3 (MD) [2–5]^c^	Screening—WST	Liquid	61/123 n = 37 (PSPRS 1–2), n = 24 (PSPRS 2–4)	PSPRS (Item 13) (0–4)	1.08 ± 1.26 (M ± SD), [0–4]; 61/123 (49.59%) (PSPRS > 0)	7/16
Warnecke et al. (2010) Germany University Hospital	Descriptive observational, cross-sectional	18	69.67 ± 8.9	11:7	3.47 ± 1.8	3 (MD) [2.5–5]^c^ 37.39 ± 13.92^b.1^	FEES	Liquid, puree, soft solids	15/18 Dysphagia score ≥ 1; 5/18 (PAS ≥ 6)	specific endoscopic dysphagia severity score (0–3, no to severe), PAS	1 (MD) (IQR 1–2) [0–3]	9/16
Johnston et al. (1997) USA Hospital	Analytic observational, case–control	7	70 (MD)	5:2	5 (MD), [3–13]	4 (MD) [3–5]^c^	PROM, VFSS, manometry	Liquid, solids	VFSS: 5/6 PROM: 6/7; VFSS: 2/6	Specific dysphagia severity score (1–7, no to severe), Penetration-Aspiration-Score (1–3, mild to severe)	PROM: 5 (MD), [1–6]; VFSS: Oral phase: 1.5 (MD) [0–2] Pharyngeal phase: 1.5 (MD) [0–3]	9/16
Alfonsi et al. (2007) Italy University and Clinic	Analytic observational, case–control	9	71 (M) [64–77]	4:5	4 [3–6]	41 (M) [29–59]^b^	PROM, EMG	Liquid	9/9	Specific dysphagia severity score based on subjective patient complaint (0–2, no to severe)	1.4 (M) [1, 2]	6/16
Litvan et al. (1997) USA Clinical Center	Analytic observational, case–control	27	64.9 ± 1.2 (SEM)	18:9	52 ± 5 months (symptom onset)	3.4 ± 0.1^c^ 2 ± 0.1^d^ 1.8 ± 0.1^b.2^	PROM, CSE, ultrasound, VFSS	Liquid, puree, solids	VFSS: 24/25 PROM: 27/27 OMS: 27/27; VFSS: 5/25	NIH-speech pathology swallowing questionnaire (SQ), Oral Motor Scale (OMS), VFSS: 4-point scale (1–4)	SQ: 6.6 ± 0.9; CSE: 2.8 ± 0.2 VFSS: Oral phase: 2.5 ± 0.2 (1–4) Pharyngeal phase: 2.8 ± 0.2 (1–4)	7/16
Sulena et al. (2017) India Tertiary Care Center	Descriptive observational, case–control	25	n/a	19:6	25 ± 8.3 months	Not specified	Screening—WST	Liquid	9/25 “Difficulty initiating swallow”	Not specified	Not specified	3/16
Golbe et al. (2020)	Analytic observational, cohort	491	71.53 ± 7.84	249:242	3.8 ± 2.63	41 ± 15.19^a^	WST	Liquid	301/491	PSPRS (Item 13) (0–4)	1 (MD), [0–3]	11/16
Piot et al. (2020) Multicenter University Clinics	Analytic Observational, cross-sectional, cohort	164	70.4 ± 7.6	62:38	3.5 ± 2.5	35.4 ± 14^a^	CROM/PROM	–	102/164	PSP-CDS, Dysphagia domain	1 (MD), [0–3]	10/16
Picillo et al. (2022)	Analytic observational, case–control	21	67.05 ± 6.31	12:9	3.14 ± 2.03	39.29 ± 15.9^a^	WST	Liquid	16/21	PSPRS (Item 13) (0–4)	2 (MD), [0–3]	7/16

Mean (M), Median (MD), [Range], ^a^PSPRS (Progressive Supranuclear Palsy Rating Scale) [58], ^b^UPDRS (Movement Disorder Society-Unified Parkinson’s Disease Rating Scale) [57], ^b.1^UPDRS III Motor Score, ^b.2^UPDRS III Item 31 Bradykinesia, ^c^H&Y (Hoehn & Yahr Scale) [59], ^d^Rafal & Grim (5 = normal, 0 = severe) [60], Dysphagia Outcome and Severity Scale (DOSS) [61], Fiberoptic Endoscopic Evaluation of Swallowing (FEES), videofluoroscopic study of swallowing (VFSS), Penetration–Aspiration Scale (PAS) [36], Modified Barium Swallowing Impairment Profile (MBSImP) [62], Functional Oral Intake Scale (FOIS) [63], Dysphagia Rating Scale (DRS) [64], oral motor scale (OMS) [65], patient-reported outcome measure (PROM), swallowing questionnaire (SQ) [66], clinical swallowing examination (CSE), electromyography (EMG), water swallowing test (WST), high-resolution manometry (HRM), Progressive Supranuclear Palsy Clinical Deficits Scale (PSP-CDS)

#### Dysphagia Assessment

Dysphagia was assessed with instrumental assessment methods in 54% of studies. Dysphagia definitions varied in specificity. Dysphagia was either defined by specific symptoms [11, 13, 15, 50, 51], additionally complemented with cut-off values based on validated scales or normative values [16, 50] or more broadly described [52]. Other studies incorporated dysphagia definitions indirectly according to the chosen assessment scale [53–55]. Different food and fluid consistencies were trialled in half of the studies, the other half provided liquid bolus only, whereas in one study it was not specified. The amounts of liquids trialled ranged from 2 to 150 ml.

#### Dysphagia Severity

The reported severity ranged from no dysphagia to severe dysphagia. Most participants seem to be affected by mild to moderate swallowing difficulties according to the reported measures of central tendency. Moreover, one study excluded severe dysphagia cases [50]. The severity of dysphagia was found to increase with disease duration, motor, and cognitive impairment [11–13, 50], but not with age [13]. Choi et al. [56] further report a significant negative correlation between dysphagia and gaze abnormalities. In contrast, Alfonsi et al. [48] state that the dysphagia scores in their study did not relate to the Movement Disorder Society-Unified Parkinson’s Disease Rating Scale (UPDRS) [57] scores but dysphagia was not assessed by the reference standard instrumental methods such as FEES or VFSS.

### Prevalence

**Fig. 2:**
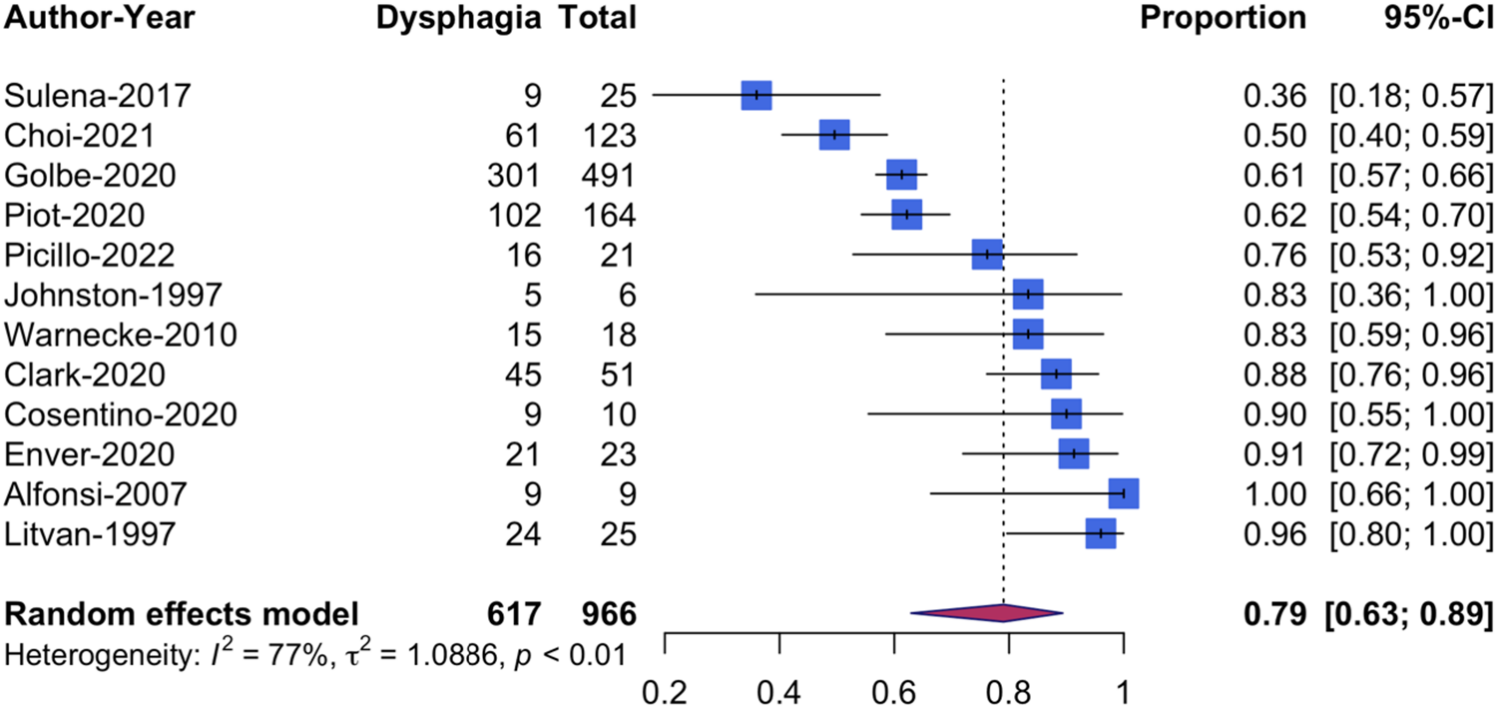
Forest plot of pooled dysphagia prevalence rates based on patient or clinician reported outcome measures, instrumental and clinical assessment

**Fig. 3:**
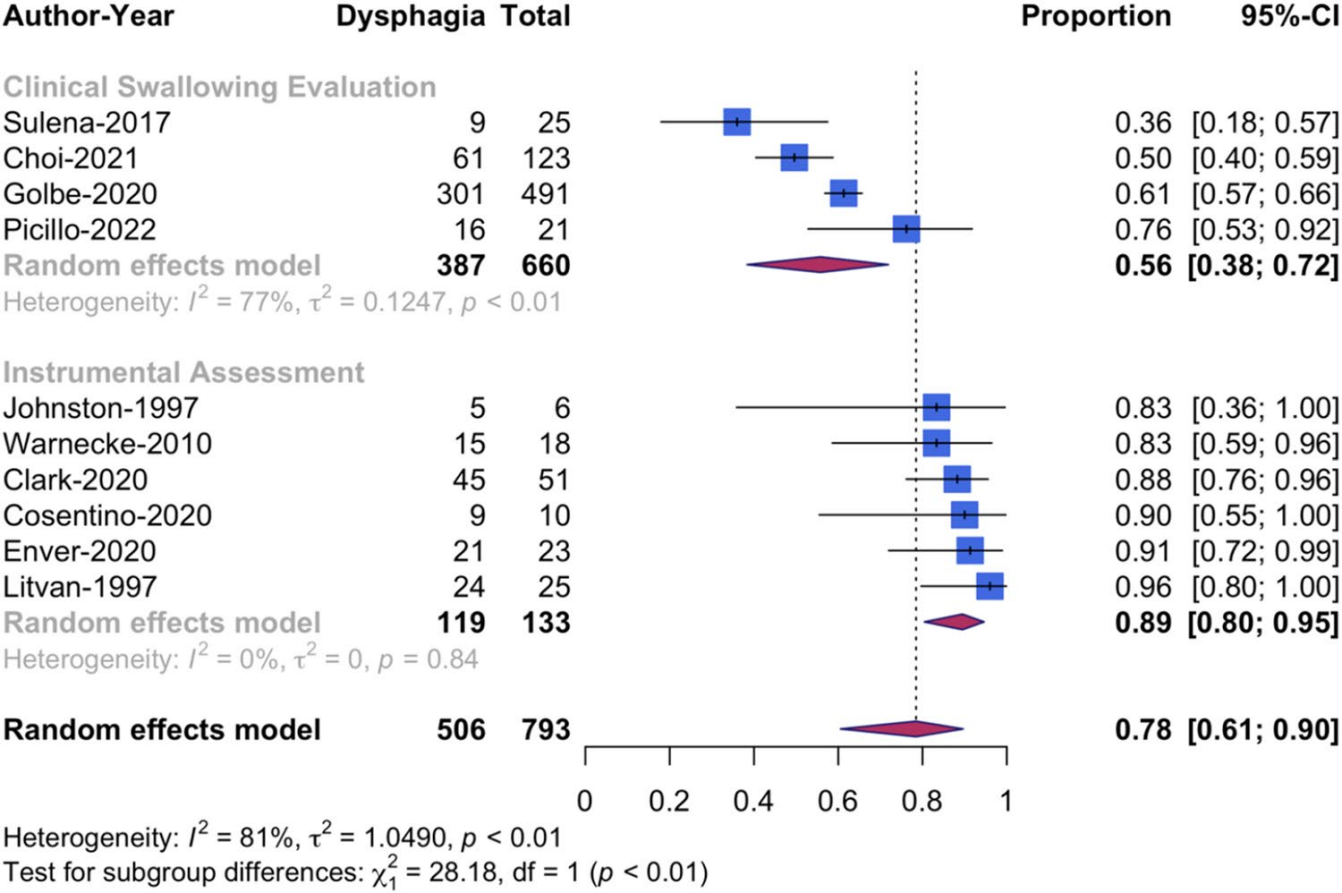
Forest plot of pooled dysphagia prevalence rates by assessment type

A random-effects model with studies weighted according to sample size was chosen (Table 2). The pooled dysphagia prevalence was 79% (CI [62.9, 89.3]) (Fig. 2). The meta-analysis showed considerable heterogeneity (*I*^2^ = 77.4%, *p* < 0.001). A subgroup analysis with studies grouped by the chosen assessment method showed considerable heterogeneity for CSE but not for instrumental assessment (Fig. 3). The prevalence rates based on PROM ranged from 86% to 100% [12, 16, 48]. However, studies based on PROM (*n* = 3) or clinician reported outcome measure (CROM) (*n* = 1) were excluded from the subgroup analysis due to the insufficient number of studies in these subgroups and/or the extreme proportion in two of three studies as it would introduce a high risk of random error or imprecision [33, 67].

**Table 2:** Meta-analysis results

Meta-analysis	Assessment	*n* of Studies	Sample size	Pooled prevalence	95% CI	*I*^2^ (%)	*χ*^2^, *p*
Dysphagia	Instrumental CSE PROM/CROM	12	966	79.4	62.9–89.3	77.4	0.001
Dysphagia	CSE	4	660	56.0	38.4–71.9	77	0.01
Dysphagia	Instrumental	6	133	89.4	80.4–94.6	0	0.84
Aspiration	Instrumental	5	123	24.1	13.6–39.0	24.95	0.26

*PROM* patient reported outcome measure, *CROM* clinician reported outcome measure, *n* number, *CI* confidence interval

**Fig. 4:**
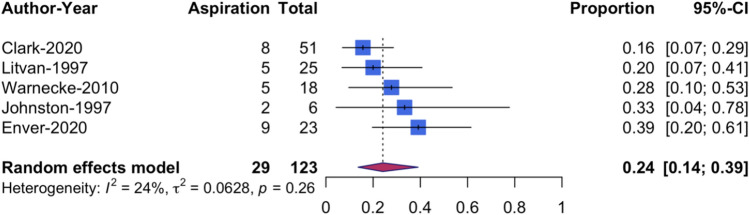
Forest plot of pooled aspiration prevalence rates based on instrumental assessment

The pooled aspiration prevalence was 24.13% (95% CI [13.6, 39.0]) (Fig. 4). The analysis showed a low variability between studies (*I*^2^ = 24.1%, *p* = 0.26). The included studies utilised either FEES or VFSS and assessed aspiration with the PAS or an equivalent scale. The pooled prevalence was only calculated for aspiration of water since this was the consistency that was trialled most frequently and the most difficult consistency to swallow for people with PSP, as indicated by studies that tested and reported different consistencies in detail [11, 13].

### Quality Assessment

**Fig. 5:**
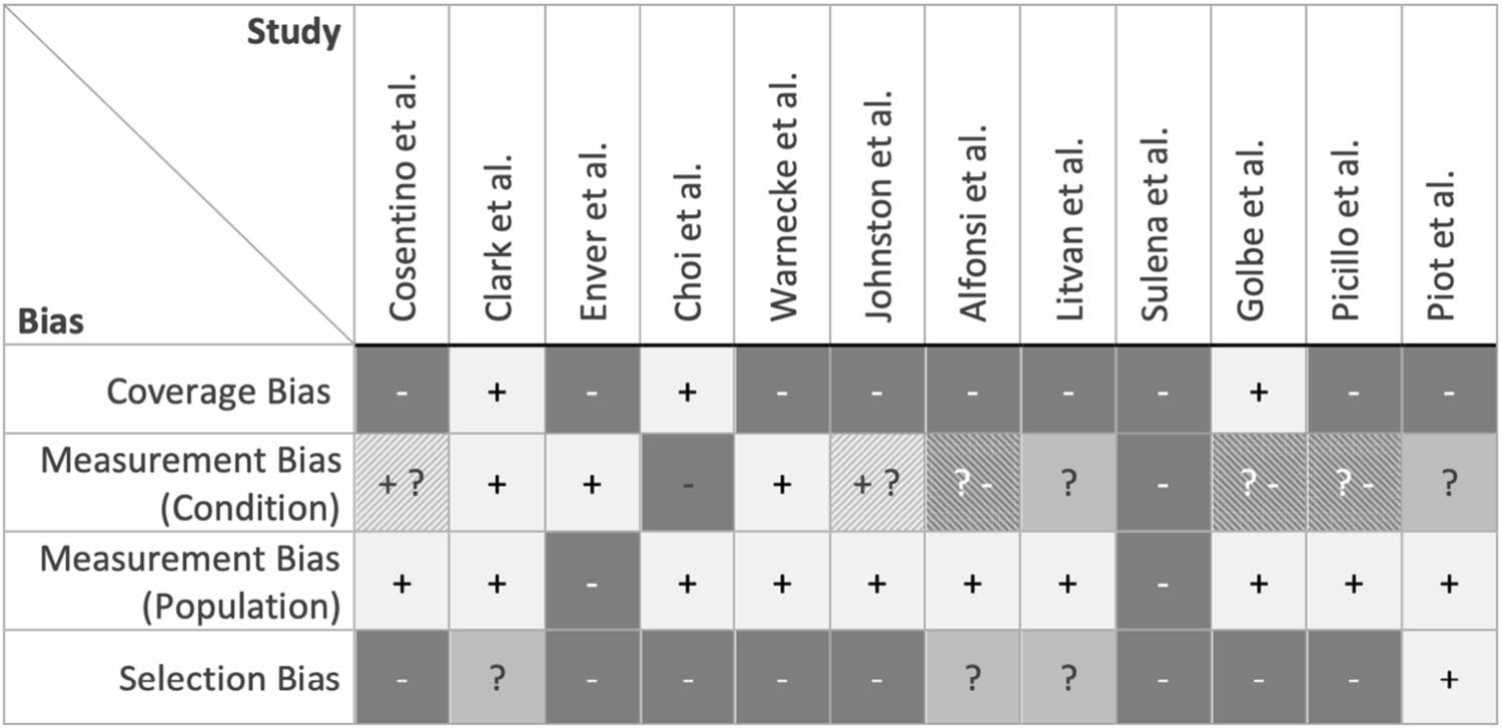
Risk of bias summary for individual studies based on the JBI Critical Appraisal Checklist for Studies Reporting Prevalence Data. Low risk of bias (+), moderate risk of bias (?), high risk of bias (−). The measurement bias of the condition is based on the items 6 and 7 of the appraisal checklist

**Fig. 6:**
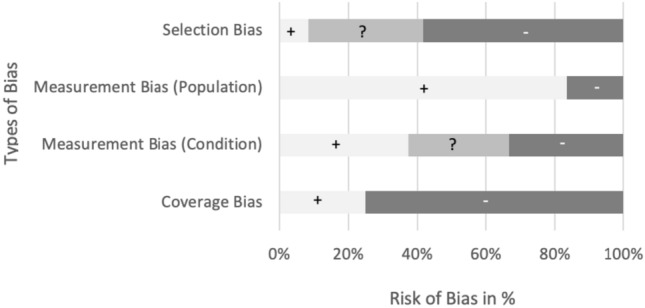
Risk of bias across studies based on the JBI Critical Appraisal Checklist for studies reporting prevalence data. Low risk of bias (+), moderate risk of bias (?), high risk of bias (−)

Using the JBI checklist for prevalence studies [32] 12 studies were assessed with a mean score of 7/16, corresponding to a moderate quality (Fig. 5). Items primarily responsible for lower quality ratings were insufficient description of the sampling strategy (*n* = 4) or convenience sampling (*n* = 8), small sample sizes (*n* = 12) and an insufficient sample coverage (*n* = 10). Insufficiently covered samples resulted from not including participants with severe dysphagia, by an uneven gender distribution as well as an overrepresentation of moderate PSP cases with a tendency towards more mild cases. Items leading to higher quality ratings were an appropriate sample frame (*n* = 8), sufficient description of participants and setting (*n* = 11), and the use of valid methods to identify dysphagia (*n* = 6). Based on the quality assessment, studies were at high risk of selection and coverage bias (Fig. 6). The measurement bias regarding the condition and population was as low to moderate in the majority of studies. An increased risk of bias resulted from unclear starting point of the reported disease duration, often missing information on interrater reliability, the description of the person/s who conducted the dysphagia assessment or their diagnostic experience. High measurement bias resulted from the implementation of dysphagia screening tools or self-created scales or missing information about the patient population.

## Discussion

This systematic review and meta-analysis found dysphagia to be highly prevalent in people with PSP. Aspiration was present in a quarter of study participants with PSP.

The pooled dysphagia prevalence of 79% (CI [62.9, 89.3]) is influenced by the chosen assessment method and ranges from 56% (CI [38.4, 71.9]) to 89% (CI [78.9, 95.0]) depending on the assessment method selected and its associated diagnostic accuracy for dysphagia and aspiration. The lower prevalence figures are reported in studies that based their dysphagia diagnosis on water-swallow tests (WST). Of note, these WST are primarily validated for use in patient with stroke [68, 69] and lack validation in people with PSP. Instrumental dysphagia assessment methods should identify impairments that might not translate to subjective symptoms of dysphagia or be detected during a dysphagia screening procedure or a clinical swallow examination (CSE). This is reflected in the meta-analysis that resulted in a higher prevalence of dysphagia when studies diagnosing dysphagia based on CSE were excluded.

The higher prevalence of dysphagia in PROM [12, 16, 48] in PSP indicates a considerable subjective impact of dysphagia on patients with PSP and is agreement with earlier literature [10]. The similarly high pooled dysphagia prevalence based on instrumental assessment corresponds to the reported strong correlations between instrumental assessment and PROM [11, 12, 16]. This suggests a high self-perception of swallowing impairment in people with PSP but again this may be an artifact of participant recruitment in studies. One study reports a lower dysphagia prevalence based on clinician reports which included younger participants in an earlier stage of PSP [55]. Nevertheless, the earlier awareness of dysphagia could distinguish PSP and IPD [70] and suggests that there may be a role for detailed PROMs in this population.

In addition to the subjective perception, the impact of the swallowing impairment is further highlighted by silent aspiration being present from 10% [11] to 34% of participants [51]. The difference between studies that report more mild dysphagia in contrast to those that report more severe swallowing impairment is a slightly longer disease duration. Thus, even though only a quarter of this sample is affected by aspiration, this figure is likely to increase. This is indicated by the reported need for tube feeding [9, 18], or dysphagia being the leading symptom in patients admitted to a palliative care unit [21].

While the findings of this systematic review are useful, it is important to note, that the prevalence figures presented here do not truly reflect people with early or late-stage PSP. The majority of participants in the included studies were moderately affected by PSP according to the indicated severity ratings of the Progressive Supranuclear Palsy Rating Scale (PSPRS) [58], the UPDRS [57], or the Hoehn & Yahr Scale [59]. Therefore included studies were deemed to be at high risk of coverage and selection bias. This overrepresentation of moderately affected patients and the mainly small samples sizes were to be expected given the general low prevalence estimate of PSP (5–6 cases per 100.000) [71–73]. Moreover, the general low prevalence of PSP impedes random sampling. It is also not surprising that only few patients with a mild impairment are included if late diagnosis [74] and frequent misdiagnosis [2] are considered. Overall, the included studies were of moderate methodological quality regarding the reporting of dysphagia prevalence with a low to moderate risk of measurement bias and a high risk of coverage and selection bias.

### Implications for Clinical Practice

There are some implications for the study findings for clinical practice. The fact that dysphagia is prevalent in PSP and progresses quickly [9, 20] suggests that dysphagia assessment ideally incorporating both PROMs and instrumental assessment should be completed early in the trajectory of the disease so that patients can be managed proactively. Any symptom of swallowing impairment in people with PSP should be identified as early as possible since dysphagia onset drastically reduces survival time [7]. Aspiration and particularly silent aspiration emphasises the need for early identification of dysphagia using instrumental assessments such as videofluoroscopy or FEES. Due to the high prevalence and rapid progression of dysphagia in this group, follow-up assessments at regular timepoints are also important in order to monitor dysphagia progression. By considering patient reports and using instrumental assessment, this could facilitate preventive, symptom-oriented intervention and thereby minimise the consequences of dysphagia. Validated PROMs on people with PSP rather than relying on those validated on other populations should be available to improve assessment.

### Strengths and Limitations of the Study

In addition to the fact that the polualtion in these studies do not represent people at both ends of the PSP disease trajectory, the size and quality of the included studies influence quantitative and qualitative data synthesis of this systematic review (SR) [42, 75]. Furthermore, the cross-sectional design of the included studies, i.e., collecting data at one point in time introduces the risk of not capturing the true severity of the examined condition [76]. It needs to be noted that the JBI checklist [32] used for the quality assessment is suited for studies with the main aim to estimate a prevalence rate. In the included studies dysphagia prevalence was not the primary aim which influences the quality rating. However, alternatively applying rating scales according to the underlying study design would have impeded comparability of the individual quality ratings and thus the overall bias assessment with regards to the aim of this study.

### Future Research

This study focused on the overall condition of PSP rather than its subcategories. Future research should explore dysphagia profiles and prevalence within these specific categories. Prospective research should also focus on the development of dysphagia in the course of PSP using instrumental assessment methods. Considering all phases of swallowing as well as the subjective impact of dysphagia on affected people would help improve quality of life for this population. Using either a cross-sectional design with more balanced samples, including more participants in early and late stages of PSP to better display dysphagia prevalence at different stages of PSP, or longitudinal studies to depict the development of dysphagia over time and its manifestation in the different phases of PSP. This would also allow for the observation of risk factors leading to swallowing difficulties.

## Conclusion

Dysphagia is highly prevalent in this sample of participants with predominantly moderate severity of PSP. An equally high dysphagia prevalence in PROM highlights its impact on people with PSP. Aspiration occurs in a quarter of this sample and is likely to increase as the disease progresses. Early carefully planned management is required.

## Other Information

### Deviation from Study Protocol

There were four deviations from the study protocol. Firstly, the inclusion criteria regarding the population, study type and languages were restricted. This was due to the amount of data produced in the systematic database search and the available time for this project, the inadequacy of abstracs as a basis for quality ratings, and missing English versions of Chinese or Japanese records. Secondly, studies were excluded after two unsuccessful contact attempts instead of being marked as ‘waiting for assessment’. Thirdly, data extraction and quality assessment were carried out by one reviewer. Lastly, due to the overlap with another project and to enhance clarity this paper was restricted to the prevalence of dysphagia in PSP. The search was updated during the course of the other project. This new search produced an overall greater number of initial records, but did not change the final number of studies to be included in this systematic review.

## Supplementary Information

Below is the link to the electronic supplementary material.

Supplementary file1 (PDF 403 kb)

## Data Availability

Data that support the results reported in this article are available in the supplementary information, this comprises the PRISMA checklist, the search strategies for all databases, excluded studies with reasons and the data items that were sought.
