# Supplementary material for: Dysphagia Prevalence in Progressive Supranuclear Palsy: A Systematic Review and Meta-Analysis
Source: Dysphagia. 2024 Mar 24;39(6):1052–64. doi: 10.1007/s00455-024-10681-7 (PMC11607008; doi:10.1007/s00455-024-10681-7)
Supplement: Supplementary file 1 — Supplementary file1 (PDF 403 kb) [file 455_2024_10681_MOESM1_ESM.pdf]

Glinzer, Flynn, Tampoukari, Harpur, Walshe; Trinity College Dublin, walshema@tcd.ie

SUPPLEMENTARY INFORMATION

PRISMA Checklist

Section and Topic	Item #	Checklist item	Location where item is reported
**TITLE**			
Title	1	Identify the report as a systematic review.	Title, Abstract, p. 2
**ABSTRACT**			
Abstract	2	See the PRISMA 2020 for Abstracts checklist.	p. 1
**INTRODUCTION**			
Rationale	3	Describe the rationale for the review in the context of existing knowledge.	p. 2
Objectives	4	Provide an explicit statement of the objective(s) or question(s) the review addresses.	p. 2
**METHODS**			
Eligibility criteria	5	Specify the inclusion and exclusion criteria for the review and how studies were grouped for the syntheses.	p.3
Information sources	6	Specify all databases, registers, websites, organisations, reference lists and other sources searched or consulted to identify studies. Specify the date when each source was last searched or consulted.	p.3
Search strategy	7	Present the full search strategies for all databases, registers and websites, including any filters and limits used.	Supplementary Information
Selection process	8	Specify the methods used to decide whether a study met the inclusion criteria of the review, including how many reviewers screened each record and each report retrieved, whether they worked independently, and if applicable, details of automation tools used in the process.	p. 3-4
Data collection process	9	Specify the methods used to collect data from reports, including how many reviewers collected data from each report, whether they worked independently, any processes for obtaining or confirming data from study investigators, and if applicable, details of automation tools used in the process.	p. 3-4
Data items	10a	List and define all outcomes for which data were sought. Specify whether all results that were compatible with each outcome domain in each study were sought (e.g. for all measures, time points, analyses), and if not, the methods used to decide which results to collect.	Supplementary Information, p. 3-4
	10b	List and define all other variables for which data were sought (e.g. participant and intervention characteristics, funding sources). Describe any assumptions made about any missing or unclear information.	Supplementary Information, p. 3-4
Study risk of bias assessment	11	Specify the methods used to assess risk of bias in the included studies, including details of the tool(s) used, how many reviewers assessed each study and whether they worked independently, and if applicable, details of automation tools used in the process.	p. 4
Effect measures	12	Specify for each outcome the effect measure(s) (e.g. risk ratio, mean difference) used in the synthesis or presentation of results.	p. 4
Synthesis methods	13a	Describe the processes used to decide which studies were eligible for each synthesis (e.g. tabulating the study intervention characteristics and comparing against the planned groups for each synthesis (item #5)).	p. 4
	13b	Describe any methods required to prepare the data for presentation or synthesis, such as handling of missing summary statistics, or data conversions.	-

Section and Topic	Item #	Checklist item	Location where item is reported
	13c	Describe any methods used to tabulate or visually display results of individual studies and syntheses.	p. 4
	13d	Describe any methods used to synthesize results and provide a rationale for the choice(s). If meta-analysis was performed, describe the model(s), method(s) to identify the presence and extent of statistical heterogeneity, and software package(s) used.	p. 4
	13e	Describe any methods used to explore possible causes of heterogeneity among study results (e.g. subgroup analysis, meta-regression).	p. 4
	13f	Describe any sensitivity analyses conducted to assess robustness of the synthesized results.	-
Reporting bias assessment	14	Describe any methods used to assess risk of bias due to missing results in a synthesis (arising from reporting biases).	-
Certainty assessment	15	Describe any methods used to assess certainty (or confidence) in the body of evidence for an outcome.	-
**RESULTS**			
Study selection	16a	Describe the results of the search and selection process, from the number of records identified in the search to the number of studies included in the review, ideally using a flow diagram.	p. 5
	16b	Cite studies that might appear to meet the inclusion criteria, but which were excluded, and explain why they were excluded.	p. 5
Study characteristics	17	Cite each included study and present its characteristics.	p. 7-8/ Table 2
Risk of bias in studies	18	Present assessments of risk of bias for each included study.	p. 11/ Fig. 5, 6
Results of individual studies	19	For all outcomes, present, for each study: (a) summary statistics for each group (where appropriate) and (b) an effect estimate and its precision (e.g. confidence/credible interval), ideally using structured tables or plots.	p. 7-8/ Table 2
Results of syntheses	20a	For each synthesis, briefly summarise the characteristics and risk of bias among contributing studies.	p. 11
	20b	Present results of all statistical syntheses conducted. If meta-analysis was done, present for each the summary estimate and its precision (e.g. confidence/credible interval) and measures of statistical heterogeneity. If comparing groups, describe the direction of the effect.	p. 9-10
	20c	Present results of all investigations of possible causes of heterogeneity among study results.	p. 9-10
	20d	Present results of all sensitivity analyses conducted to assess the robustness of the synthesized results.	p. 10
Reporting biases	21	Present assessments of risk of bias due to missing results (arising from reporting biases) for each synthesis assessed.	p. 11-12
Certainty of evidence	22	Present assessments of certainty (or confidence) in the body of evidence for each outcome assessed.	-
**DISCUSSION**			
Discussion	23a	Provide a general interpretation of the results in the context of other evidence.	p. 12-13
	23b	Discuss any limitations of the evidence included in the review.	p. 14
	23c	Discuss any limitations of the review processes used.	p. 14
	23d	Discuss implications of the results for practice, policy, and future research.	p. 14-15
**OTHER INFORMATION**			
Registration and protocol	24a	Provide registration information for the review, including register name and registration number, or state that the review was not registered.	Title page

Section and Topic	Item #	Checklist item	Location where item is reported
	24b	Indicate where the review protocol can be accessed, or state that a protocol was not prepared.	Title page
	24c	Describe and explain any amendments to information provided at registration or in the protocol.	p. 15
Support	25	Describe sources of financial or non-financial support for the review, and the role of the funders or sponsors in the review.	Title page
Competing interests	26	Declare any competing interests of review authors.	Title page
Availability of data, code and other materials	27	Report which of the following are publicly available and where they can be found: template data collection forms; data extracted from included studies; data used for all analyses; analytic code; any other materials used in the review.	Title page

From: Page MJ, McKenzie JE, Bossuyt PM, Boutron I, Hoffmann TC, Mulrow CD, et al. The PRISMA 2020 statement: an updated guideline for reporting systematic reviews. BMJ 2021;372:n71. doi: 10.1136/bmj.n71

MOOSE Checklist for Meta-analyses of Observational Studies (Stroup et al., 2000)

Item No	Recommendation	Reported on Page No
Reporting of background should include		
1	Problem definition	2
2	Hypothesis statement	-
3	Description of study outcome(s)	3-4
4	Type of exposure or intervention used	-
5	Type of study designs used	2-3
6	Study population	3
Reporting of search strategy should include		
7	Qualifications of searchers (eg, librarians and investigators)	3
8	Search strategy, including time period included in the synthesis and key words	3 Supplementary information
9	Effort to include all available studies, including contact with authors	3-4
10	Databases and registries searched	3
11	Search software used, name and version, including special features used (eg, explosion)	3
12	Use of hand searching (eg, reference lists of obtained articles)	3
13	List of citations located and those excluded, including justification	Supplementary information
14	Method of addressing articles published in languages other than English	-
15	Method of handling abstracts and unpublished studies	3, 15
16	Description of any contact with authors	4
Reporting of methods should include		
17	Description of relevance or appropriateness of studies assembled for assessing the hypothesis to be tested	4
18	Rationale for the selection and coding of data (eg, sound clinical principles or convenience)	-
19	Documentation of how data were classified and coded (eg, multiple raters, blinding and interrater reliability)	4, Supplementary information
20	Assessment of confounding (eg, comparability of cases and controls in studies where appropriate)	4

21	Assessment of study quality, including blinding of quality assessors, stratification or regression on possible predictors of study results	4
22	Assessment of heterogeneity	4
23	Description of statistical methods (eg, complete description of fixed or random effects models, justification of whether the chosen models account for predictors of study results, dose-response models, or cumulative meta-analysis) in sufficient detail to be replicated	4
24	Provision of appropriate tables and graphics	See tables and figures
Reporting of results should include		
25	Graphic summarizing individual study estimates and overall estimate	Fig. 2
26	Table giving descriptive information for each study included	Table 1
27	Results of sensitivity testing (eg, subgroup analysis)	Fig. 3
28	Indication of statistical uncertainty of findings	12-13
Reporting of discussion should include		
29	Quantitative assessment of bias (eg, publication bias)	Figure 5, 6; 11-12
30	Justification for exclusion (eg, exclusion of non-English language citations)	Supplementary Material; p.5
31	Assessment of quality of included studies	Table 1, 11-12
Reporting of conclusions should include		
32	Consideration of alternative explanations for observed results	13-14
33	Generalization of the conclusions (ie, appropriate for the data presented and within the domain of the literature review)	13-15
34	Guidelines for future research	14
35	Disclosure of funding source	Title page

From: Stroup DF, Berlin JA, Morton SC, et al, for the Meta-analysis Of Observational Studies in Epidemiology (MOOSE) Group. Meta-analysis of Observational Studies in Epidemiology. A Proposal for Reporting. *JAMA*. 2000;283(15):2008-2012. doi: 10.1001/jama.283.15.2008.

Search Strategies

Search Strategy CINAHL (04-2021)
**#1 Search String regarding Population**
((MH "Supranuclear Palsy, Progressive") OR TI progressive supranuclear palsy OR AB progressive supranuclear palsy OR AB steele-richardson-olszewski-syndrome OR TI steele-richardson-olszewski-syndrome OR AB richardson syndrome OR TI richardson syndrome)
**#2 Search-Term regarding Condition**
((MH "Deglutition Disorders") OR (MH "Deglutition") OR (MH "Eating") OR (MH "Salivation") OR (MH "Mastication") ORTI swallow* OR AB swallow* OR TI dysphagi* OR AB dysphagi*)
#1 AND #2

Search Strategy Web of Science (04-2021)
**#1 Search String regarding Population**
(TI=(progressive supranuclear palsy) OR AB=(progressive supranuclear palsy) OR TI=(Steele Richardson Olszewski) OR AB=(Steele Richardson Olszewski) OR TI=(richardson syndrome) OR AB=(richardson sndrome) OR TI=(Progressive Supranuclear Ophthalmoplegia) OR AB=(Progressive Supranuclear Ophthalmoplegia))
**#2 Search-Term regarding Condition**
(TI=(deglutition) OR AB=(deglutition) OR TI=(swallow*) OR AB=(swallow*) OR TI=(dysphagi*) OR AB=(dysphagi*) OR TI=(feeding disorder*) OR AB=(feeding disorder*) OR TI=(drinking disorder*) OR AB=(drinking disorder*))
#1 AND #2

Search Strategy Embase (04-2021)
**#1 Search String regarding Population**

'progressive supranuclear palsy'/exp OR ‘steele richardson olszewski syndrome’:ab,ti OR 'richardson syndrome':ab,ti OR 'progressive supranuclear ophthalmoplegia':ab,ti
**#2 Search-Term regarding Condition**
'dysphagia'/exp OR 'swallowing'/exp OR deglutition*:ti,ab OR swallow*:ti,ab OR dysphagi*:ti,ab OR 'feeding disorder'/exp OR 'drinking disorder*':ti,ab
#1 AND #2

Search Strategy ProQuest Dissertations & Theses (04-2021)
**#1 Search String regarding Population**
ti("progressive supranuclear palsy") OR ab("progressive supranuclear palsy") OR "steele-richardson-olszewski syndrome" OR "richardson syndrome"
**#2 Search-Term regarding Condition**
ti(deglutition) OR ab(deglutition) OR ti("eating disorder*") OR ab("eating disorder*") OR ti("Dysphagia") OR ab("Dysphagia") OR ti(swallow*) OR ab(swallow*)
#1 AND #2

Search Strategy OpenGrey (04-2021)
**#1 Search String regarding Population**
“progressive supranuclear palsy”
**#2 Search-Term regarding Condition**
(deglutition* OPT disorder*) OR (Swallow* OPT impair*) OR (Dysphagia)
#1 AND #2

Search Strategy Pubmed (04-2021)
**#1 Search String regarding Population**
Supranuclear Palsy, Progressive[Mesh] OR Steele Richardson Olszewski [Title/Abstract] OR Richardson Syndrome[Title/Abstract] OR Richardson's Syndrome[Title/Abstract] OR Progressive Supranuclear Ophthalmoplegia[Title/Abstract]
**#2 Search-Term regarding Condition**
Deglutition Disorders[Mesh] OR deglutition[Mesh] OR swallow*[Title/Abstract] OR dysphagi*”[Title/Abstract] OR deglutition*” [Title/Abstract] OR feeding disorder*” [Title/Abstract] OR drinking disorder*” [Title/Abstract]
#1 AND #2

Exemplary Updated Search Strategy Pubmed (04-2022)
**#1 Search String regarding Population**
Supranuclear Palsy, Progressive[Mesh] OR supranuclear pals*[Title/Abstract] OR PSP[Title/Abstract] OR richardson syndrome*[Title/Abstract]
**#2 Search-Term regarding Condition**
Deglutition Disorders[Mesh] OR deglutition*[Title/Abstract] OR dysphagi*[Title/Abstract] OR swallow*[Title/Abstract]
#1 AND #2

Data Items

	Outcome	Definition
**General**	Covidence #	N/S
	Study ID	First Author, Year of Publication
	Title	N/S
	Lead Author	N/S
	Contact Details	N/S
	DOI	N/S
	Year of Publication	N/S
	Eligibility	

**Context**	Country	N/S
	Setting	N/S
**Study Characteristics**	Aim of the Study	N/S
	Timeline	Either prospective or retrospective
	Study Design<br>• Observational<br>o Analytic observational<br>o Descriptive observational<br>• Experimental	N/S
	Funding Sources	N/S
	Possible Conflicts of Interest	N/S
	Number of Participants	N/S
**Population**	Inclusion Criteria	N/S
	Exclusion Criteria	N/S
	Diagnosed by a neurologist/ adherence to universally accepted diagnostic criteria	N/S
	Assessment Tool	N/S
	Age	N/S
	Sex	N/S
	Disease Duration	N/S
	Disease Severity	As indicated by chosen assessment tool
	Co-Morbidities	N/S
	Survival time	N/S
	Cause of death (if applicable)	N/S
**Condition**	Definition	N/S
	Type of Assessment	N/S
	Assessment Tool/Rating Scale/ Instrument	N/S
	Severity	As indicated by chosen assessment tool(s)
	Prevalence rate of Dysphagia	Proportion of number of participants that presented with impaired swallowing according to the chosen assessment tool/rating scale/ instrument divided by the number of participants of each study
	Prevalence rate of Aspiration	Proportion of number of participants that presented with impaired swallowing according PAS score ≥ 6 or an equal value of another scale and needed to be assessed by either FEES or VFSS divided by the number of participants of each study

Excluded full-text articles with reasons

Authors	Year	Title	Reason for Exclusion
Mahale, R. R.; Krishnan, S.; Divya, K. P.; Jisha, V. T.; Kishore, A.	2022	Gender differences in progressive supranuclear palsy	Insufficient Information about Condition
Borders, J. C.; Curtis, J. A.; Sevitz, J. S.; Vanegas-Arroyave, N.; Troche, M. S.	2022	Immediate Effects of Sensorimotor Training in Airway Protection (smTAP) on Cough Outcomes	Overlap of participants

		in Progressive Supranuclear Palsy: A Feasibility Study	
Natera-Villalba, E.; Martinez-Castrillo, J. C.; Moreno, Jlls; Gomez-Lopez, A.; Sanchez-Sanchez, A.; Lopez-Martinez, M. J.; Rabano, A.; Alonso-Canovas, A.	2022	Eye-of-the-Tiger Sign with an Unexpected Pathological Diagnosis	Insufficient Information about Condition
Maduri, P.; Mucha, C.; Bushi, S.	2022	Hypo-arousal Secondary to Frontotemporal Involvement in Progressive Supranuclear Palsy (PSP): A Case Report	Unable to obtain full text
Borders, J. C.; Sevitz, J. S.; Curtis, J. A.; Vanegas-Arroyave, N.; Troche, M. S.	2021	Sensorimotor Cough Dysfunction Is Prevalent and Pervasive in Progressive Supranuclear Palsy	Wrong Outcomes
Clark, H. M.; Tosakulwong, N.; Weigand, S. D.; Ali, F.; Botha, H.; Pham, N. T. T.; Schwarz, C. G.; Reid, R. I.; Senjem, M. L.; Jack, C. R., Jr.; Lowe, V. J.; Ahlskog, J. E.; Josephs, K. A.; Whitwell, J. L.	2021	Gray and White Matter Correlates of Dysphagia in Progressive Supranuclear Palsy	Overlap of participants
De La Paz, A.; Drickamer, M.	2021	The eyes have it; progressive supranuclear palsy at a glance	Unable to obtain full text
Mahale, R. R.; Krishnan, S.; Divya, K. P.; Jisha, V. T.; Kishore, A.	2021	Subtypes of PSP and Prognosis: A Retrospective Analysis	Insufficient Information about Condition
Morgan, J. C.; Ye, X.; Mellor, J. A.; Golden, K. J.; Zamudio, J.; Chiodo, L. A.; Bao, Y.; Xie, T.	2021	Disease course and treatment patterns in progressive supranuclear palsy: A real-world study	Insufficient Information about Condition
Nozaki, S.; Fujiu-Kurachi, M.; Tanimura, T.; Ishizuka, K.; Miyata, E.; Sugishita, S.; Imai, T.; Nishiguchi, M.; Furuta, M.; Yorifuji, S.	2021	Effects of Lee Silverman Voice Treatment (LSVT LOUD) on Swallowing in Patients with Progressive Supranuclear Palsy: A Pilot Study	Wrong Outcomes
Nozaki, S.; Fujiu-Kurachi, M.; Tanimura, T.; Ishizuka, K.; Miyata, E.; Sugishita, S.; Imai, T.; Nishiguchi, M.; Furuta, M.; Yorifuji, S.	2021	Effects of Lee Silverman Voice Treatment (LSVT LOUD) on Swallowing in Patients with Progressive Supranuclear Palsy: A Pilot Study	Wrong Outcomes
Nübling, G. S.; Butzhammer, E.; Lorenzl, S.	2021	Assisted Suicide in Parkinsonian Disorders	Insufficient Information about Condition
Megherbi, L.; Si Ahmed, H.; Daoudi, S.	2021	Unusual progressive supranuclear Palsy: A case report	Unable to obtain full text
Neji, A.; Nasri, A.; Sghaier, I.; Mrabet, S.; BenDjebara, M.; Kacem, I.; Gouider, R.	2021	Predictors of survival in Progressive Supranuclear Palsy in a Tunisian cohort	Unable to obtain full text
Shepherd, G.; Wongwarawipat, T.; Chatterjee, A.	2021	A retrospective review of clinical features of Progressive Supranuclear Palsy (PSP) in a movement disorders clinic	Exclusion reason: Unable to obtain full text;
Viscidi, E.; Litvan, I.; Dam, T.; Juneja, M.; Li, L.; Krzywy, H.; Eaton, S.; Hall, S.; Kupferman, J.; Höglinger, G. U.	2021	Clinical Features of Patients With Progressive Supranuclear Palsy in an US Insurance Claims Database	Insufficient Information about Condition
Alster, P.; Madetko, N.; Koziorowski, D.; Friedman, A.	2020	Progressive Supranuclear Palsy-Parkinsonism Predominant (PSP-P)-A Clinical Challenge at the Boundaries of PSP and Parkinson's Disease (PD)	Wrong Study Design
Bateman, Michelle; Parveen, Sabiha; Brickell, Brandt; Romoser, Chad; Passmore, Tim	2020	Effects of recreational therapy and speech therapy among participants with Parkinson disease and Parkinson plus conditions: Findings from a 16-week multidisciplinary program	Insufficient Information about Condition
Brown, L.; Oswal, M.; Samra, A. D.; Martin, H.; Burch, N.; Colby, J.; Lindahl, A.; Skelly, R.	2020	Mortality and Institutionalization After Percutaneous Endoscopic Gastrostomy in Parkinson's Disease and Related Conditions	Insufficient Information about Condition
Ibilah, T.; Saenz, J.J.D.; Gavito-Higuera, J.; Salazar, R.	2020	Progressive supranuclear palsy: Improved cognitive-behavioral disturbances and function of motor disabilities with antidepressants and cholinesterase inhibitors	Wrong Study Design
Ioan, P.A.; Ioghen, O.C.; Ribigan, A.-C.; Antochi, F.; Bajenaru, O.A.	2020	A possible link between Cisplatin treatment and tau pathology (an overlap between Progressive Supranuclear Palsy and Frontotemporal Degeneration)	Unable to obtain full text
Nozaki, S.; Nishiguchi, M.; Takeiti, M.; Nakano, Y.	2020	Telerehabilitation for patients with dysphagia	Unable to obtain full text
Shilimkar, Y.; Londhe, C.; Sundar, U.; Darole, P.	2020	Dysphagia in Parkinsonism: Prevalence, Predictors and Correlation with Severity of Illness Fasting)	Unable to obtain full text
Ivaniuk, A.; Solodovnikova, Y.; Marusich, T.	2020	Progressive supranuclear palsy initially misdiagnosed as Hashimoto encephalopathy	Unable to obtain full text
Bonnin, J.; Wasserman, E.; Kapogiannis, D.; Garringer, H.; Richardson, R.; Epperson, F.; Grafman, J.; Ghetti, B.	2019	Progressive supranuclear palsy with severe spinal cord involvement, presenting as frontotemporal lobar degeneration and motor neuron disease	Wrong patient population
Brown, L.; Oswal, M.; Martin, H.; Lindahl, A.; Skelly, R.	2019	Outcomes of percutaneous endoscopic gastrostomy (PEG) feeding in Parkinson's	Insufficient Information about Condition

Curtis, J.; Sevitz, J.; Perry, S.E.; Vanegas, N.; Dakin, A.; Troche, M.S.	2019	Comparison of voluntary cough effectiveness between aspirators and non-aspirators in progressive supranuclear palsy	Unable to obtain full text
Gupta, H.	2019	Bilateral INO in PSP	Insufficient Information about Condition
Ishihara, T.; Oeda, T.; Tomita, S.; Umemura, A.; Kohsaka, M.; Park, K.; Tahara, M.; Yamamoto, K.; Sawada, H.	2019	Ocular Motor Dysfunction as the Predictable Milestone of Life Prognosis in Progressive Supranuclear Palsy	Unable to obtain full text
Kaiyrzhanov, R.; Taskinbayeva, A.; Shashkin, C.	2019	Hydrocephalic presentation of PSP and follow-up after ventricularperitoneal shunting: Case report	Unable to obtain full text
Kumar, D.; Bachan, M.; Khan, Z.; Belok, T.	2019	A CASE OF SEVERE HYPERNATREMIA IN A PATIENT WITH PROGRESSIVE SUPRANUCLEAR PALSY	Wrong Study Design
Miki, Y.; Foti, S. C.; Asi, Y. T.; Tsushima, E.; Quinn, N.; Ling, H.; Holton, J. L.	2019	Improving diagnostic accuracy of multiple system atrophy: a clinicopathological study	Unable to obtain full text
Miki, Y.; Ling, H.; Foti, S.; Holton, J.	2019	Clinical and pathological features of multiple system atrophy and multiple system atrophy look-alikes	Unable to obtain full text
Miller-Patterson, C.; Burton, E.	2019	Neuromuscular electrical stimulation for the treatment of jaw-closing dystonia	Unable to obtain full text
Nogawa, S.; Chin, Y.; Kanke, H.; Kawamura, R.; Nakayama, T.; Tokuoka, K.; Furukawa, T.	2019	Evaluation of swallowing function by video-fluorography in patients with Parkinsonism	Unable to obtain full text
Nozaki, S.; Tanimura, T.; Fujiu-Kurachi, M.; Nishiguchi, M.; Furuta, M.; Yorifuji, S.	2019	In advanced Parkinsonism, Lee Silverman Voice Treatment is effective for both swallowing and speech	Unable to obtain full text
Oki, T.; Sugawara, N.; Haruta, H.; Takanashi, J.	2019	The educational programs for extrapyramidal disease and spinocerebellar degeneration patients and their families	Insufficient Information about Condition
Perry, S.E.; Sevitz, J.; Curtis, J.A.; Vanegas, N.; Seikaly, Z.; Troche, M.S.	2019	Differences in swallowing and cough function in progressive supranuclear palsy and Parkinson's disease	Unable to obtain full text
Piot, I.; Schweyer, K.; Respondek, G.; Grimm, M.; Stamelou, M.; Sckopke, P.; Schenk, T.; Goetz, C.; Stebbins, G.; Höglinger, G.	2019	The Progressive Supranuclear Palsy Functional Disability Scale: Sensitivity to change	Unable to obtain full text
Katoh, M.; Ueha, R.; Sato, T.; Sugasawa, S.; Goto, T.; Yamauchi, A.; Yamasoba, T.	2019	Choice of Aspiration Prevention Surgery for Patients With Neuromuscular Disorders: Report of Three Cases	Wrong patient population
Sebastian, R.; Gray, W.; Foley, A.; Trendall, L.; Hand, A.; Oh, D.; Dodds, S.; McLenaghan, E.; Dossantos, V.; Oates, L.; McDonald, C.; Walker, R.	2019	What factors predict hospital admissions in communitydwelling people with Parkinson's?	Unable to obtain raw data
Shea, Y. F.; Shum, A. C. K.; Lee, S. C.; Chiu, P. K. C.; Leung, K. S.; Kwan, Y. K.; Mok, F. C. K.; Chan, F. H. W.	2019	Natural clinical course of progressive supranuclear palsy in Chinese patients in Hong Kong	Insufficient Information about Condition
Soliman, D.Y.; Alhashel, J.; Kamal, W.; Youssry, D.	2019	Botulinum toxin A for sialorrhoea in parkinsonian disorders: Impact on drooling frequency and severity scale	Unable to obtain full text
Tan, C.C.	2019	A case of progressive supranuclear palsy from the geriatric perspective	Unable to obtain full text
Thongchuam, Y.; Panyakaew, P.; Bhidayasiri, R.	2019	Oromandibular manifestations in Parkinson's disease and atypical parkinsonian disorders: A video case-series analysis	Wrong Outcomes
Viscidi, E.; Zabar, Y.; Dam, T.; Juneja, M.; Kupferman, J.; Kupelian, V.; Eaton, S.; Litvan, I.; Höglinger, G.	2019	Development of a predictive model for progressive supranuclear palsy using real world data	Unable to obtain full text
Xie, T.; Ye, X.; De Courcy, J.; Mellor, J.; Zamudio, J.; Chiodo, L.; Bao, Y.	2019	The Impact of Progressive Supranuclear Palsy (PSP) Disease Severity on Healthcare Resource Utilization (HCRU)	Unable to obtain full text
Yamamoto, T.	2019	Relationship between aspiration and impaired cough reflex in parkinsonian syndrome	Unable to obtain full text
Alsomali, H.; O'Mara, G.	2018	A curious case of Parkinsonism	Unable to obtain full text
Beschin, Nicoletta; Reverberi, Cristina; Della Sala, Sergio	2018	Anosognosia for chronic dysphagia	Wrong Study Design
Chen, S.; Jakab, I.; Zelei, T.; Szilberhorn, L.; Bendes, R.; Elezbawy, B.; Kalv≥, Z.; Mann, M.; Potashman, M.; Pitter, J.	2018	Burden of progressive supranuclear palsy: A systematic literature review	Insufficient Information about Condition
Clarke, G.; Fistein, E.; Holland, A.; Tobin, J.; Barclay, S.; Barclay, S.	2018	Planning for an uncertain future in progressive neurological disease: a qualitative study of patient and family decision-making with a focus on eating and drinking	Insufficient Information about Condition

Claus, I.; Suttrup, J.; Muhle, P.; Suntrup-Krueger, S.; Siemer, M. L.; Lenze, F.; Dziewas, R.; Warnecke, T.	2018	Subtle Esophageal Motility Alterations in Parkinsonian Syndromes: Synucleinopathies vs. Tauopathies	Unable to obtain raw data
Crosiers, D.; Sieben, A.; Baets, J.; Martin, J.J.; Cras, P.	2018	Cerebellar signs in progressive supranuclear palsy	Insufficient Information about Condition
Holla, V.; Lenka, A.; Stezin, A.; Prasad, S.; Kamble, N.; Yadav, R.; Pal, P.	2018	Comparative study of non-motor symptoms in patients with progressive supranuclear palsy and age, gender, duration of illness matched patients with Parkinson's disease	Unable to obtain full text
Lewis, C.; Vogel, A.; Walterfang, M.; Velakoulis, D.	2018	Review of mealtime difficulties following frontotemporal lobar degeneration	Wrong Study Design
Lutfallah, A.A.; Dagher, C.; Naccache, N.; Yazbeck, P.	2018	Anaesthetic management in a patient with progressive supranuclear palsy	Insufficient Information about Condition
Nozaki, S.; Fujiu-Kurachi, M.; Furuta, M.; Yorifuji, S.	2018	Effect of lee silverman voice treatment (LSVT¬Æ LOUD) on dysphagia in patients with parkinsonism (progressive supranuclear palsy and multiple system atrophy)	Unable to obtain full text
Tandra, S.; Mridula, R.M.; Borgohain, R.	2018	Blink Recovery Pattern and Somatosensory Evoked Blink Reflex in patients with Parkinsonism syndromes	Unable to obtain full text
Jonnalagadda, M.	2018	Blink recovery pattern and somatosensory-evoked blink refex in patients with Parkinson's disorders and its correlation to clinico-imageological features	Unable to obtain full text
Pell, E.	2018	Progressive supranuclear palsy: longitudinal study by acoustical analysis of speech	Insufficient Information about Condition
Potashman, M.; Zelei, T.; Bendes, R.; Jakab, I.; Szilberhorn, L.; Elezbawy, B.; Kal√≥, Z.; Mann, M.; Inuzuka, Y.; Pitter, J.G.	2018	HUMANISTIC AND ECONOMIC BURDEN OF PROGRESSIVE SUPRANUCLEAR PALSY: A SYSTEMATIC LITERATURE REVIEW	Insufficient Information about Condition
Sawal, N.; Bansal, P.; Gupta, R.	2018	Methylphenidate in Progressive Supranuclear Palsy [PSP] - Old drug, New Indications?	Unable to obtain full text
Tonan, M.; Egi, M.; Furushima, N.; Mizobuchi, S.	2018	A case of spinal anesthesia in a patient with progressive supranuclear palsy	Insufficient Information about Condition
Xie, T.; Ye, X.; Kandukuri, L.; Bao, Y.	2018	Burden among patients with progressive supranuclear palsy	Unable to obtain full text
Yokota, J.-I.; Motoi, Y.; Yamaguchi, Y.	2018	Transitory alternating saccade in a case with frontotemporal dementia caused by progressive supranuclear palsy	Insufficient Information about Condition
Aiba, I.; Ikeuchi, T.; Takigawa, H.; Shimohata, T.; Tokuda, T.; Morita, M.; Onodera, O.; Murayama, S.; Hasegawa, K.; Nakashima, K.	2017	Progression of milestones by clinical types in progressive supranuclear palsy: A longitudinal observational study of a cohort of patients with PSP/CBD (the JALPAC project)	Unable to obtain full text
Clark, H.; Whitwell, J.; Ahlskog, J.; Josephs, K.	2017	Dysphagia in PSP	Overlap of participants
de Souza, Leonardo Cruz; de Paula Fran√ßa Resende, Elisa; Magalh√£es, Daiane; Teixeira, Ant√¥nio L√∫cio; Gomez, Rodrigo Santiago	2017	Wall-eyed bilateral internuclear ophtalmoplegia (WEBINO) in a patient with Richardson's syndrome, Progressive supranuclear palsy	Unable to obtain raw data
Glasmacher, S. A.; Leigh, P. N.; Saha, R. A.	2017	Predictors of survival in progressive supranuclear palsy and multiple system atrophy: a systematic review and meta-analysis	Insufficient Information about Condition
Kim, M.-J.; Kim, H.J.; Kim, J.-K.; Na, H.-R.; Koh, S.-B.	2017	Advance care planning in advanced Parkinsonian patients in a long-term care hospital	Wrong patient population
Manabe, T.; Mizukami, K.; Akatsu, H.; Hashizume, Y.; Ohkubo, T.; Kudo, K.; Hizawa, N.	2017	Factors Associated with Pneumonia-caused Death in Older Adults with Autopsy-confirmed Dementia	Insufficient Information about Condition
Miyata, N.	2017	Progressive supranuclear palsy accompanied by spasmodic dysphonia	Unable to obtain full text
Londhe, C.; Shilimkar, Y.; Sundar, U.; Darole, P.; Gala, R.; Dumade, P.	2017	Swallowing dysfunction in parkinsonian syndromes: Prevalence and correlation with severity of illness	Unable to obtain full text
Hipp, G.; Vaillant, M.; Gantenbein, M.; Kerschenmeyer, M.; Kolber, P.; Stallinger, C.; Roomp, K.; Longhino, L.; Schweicher, A.; Kr√ºger, R.; Simons, J.	2017	The french version of the munich dysphagia test-Parkinson's disease (MDT-PD): Translation and validation in a multilingual population of the Luxembourg Parkinson's Study (HELP-PD)	Unable to obtain raw data
Clark, H.; Whitwell, J.; Ahlskog, J.; Josephs, K.	2017	Tongue strength in PSP	Unable to obtain raw data
Alfonsi, E; Restivo, DA; Cosentino, G; De Icco, R; Bertino, G; Schindler, A; Todisco, M; Fresia, M; Cortese, A; Prunetti, P; Ramusino, MC; Moglia, A; Sandrini, G; Tassorelli, C	2017	Botulinum Toxin Is Effective in the Management of Neurogenic Dysphagia. Clinical-Electrophysiological Findings and Tips on Safety in Different Neurological Disorders	Unable to obtain full text

Osaki, Y.; Morita, Y.; Miyamoto, Y.; Furuta, K.; Furuya, H.	2017	Freezing of gait is an early clinical feature of progressive supranuclear palsy	Insufficient Information about Condition
Suttrup, I.; Suntrup-Krueger, S.; Pilatus, A.; Siemer, M.; Bauer, J.; Dziewas, R.; Warnecke, T.	2017	Esophageal motor disorders in atypical parkinsonian syndromes: Synucleinopathies versus. tauopathies	Wrong study design
Tadokoro, K.; Sato, K.; Morihara, R.; Shang, J.; Takemoto, M.; Ohta, Y.; Yamashita, T.; Hishikawa, N.; Abe, K.	2017	Non-ketotic hyperosmolar coma after percutaneous endoscopic gastrostomy in an advanced stage of progressive supranuclear palsy	Insufficient Information about Condition
Trufanov, Y.	2017	Differential diagnosis of Parkinson's disease and Progressive Supranuclear Palsy	Unable to obtain full text
Yamamoto, T.; Kawasaki, H.; Furuya, T.; Miyake, A.; Mitsufuji, T.; Fukuoka, T.; Ito, Y.; Nakazato, Y.; Takahashi, K.; Tamura, N.; Araki, N.	2017	Levodopa induced dyskinesia in a case of progressive supranuclear palsy	Unable to obtain full text
Bravo, J.J.; Gallardo, M.J.; Cabello, J.P.; Ibanez, R.E.; Vaamonde, J.	2016	Apraxia of speech as the initial manifestation of progressive supranuclear palsy	Unable to obtain full text
Bukki, J.; Nübling, G.; Lorenzl, S.	2016	Managing Advanced Progressive Supranuclear Palsy and Corticobasal Degeneration in a Palliative Care Unit: Admission Triggers and Outcomes	Insufficient Information about Condition
Costa Neves, B.; Mateiro, R.	2016	Othello syndrome as a presenting manifestation of progressive supranuclear palsy	Insufficient Information about Condition
Dunlop, S. R.; Kent, V. P.; Lashley, M.; Caruana, T.	2016	The Cure PSP Care Guide: A Telephonic Nursing Intervention for Individuals and Families Living With Progressive Supranuclear Palsy	Insufficient Information about Condition
Fujioka, S.; Umemoto, G.; Fukae, J.; Tsuboi, Y.	2016	Estimated annual rate of progression of dysphagia in neurodegenerative parkinsonian disorders	Unable to obtain full text
Maetzler, W.; Rattay, T. W.; Hobert, M. A.; Synofzik, M.; Bader, A.; Berg, D.; Schaeffer, E.; Rommel, N.; Devos, D.; Bloem, B. R.; Bender, B.	2016	Freezing of Swallowing	Wrong Study Design
Galvin, J.	2016	Dimensions and predictors of perceived burden among caregivers of patients with frontotemporal degeneration	Wrong Outcomes
Lorenzl, S.; Richinger, C.; Schmotz, C.; Nübling, G.	2016	Best supportive care or palliative care for late stage Parkinsonian syndromes?	Unable to obtain full text
Shirazi-Nejad, A.; Chappell, A.; Rezwan, N.; Tun, G.S.Z.; Kapur, K.; Soliman, A.; Atkinson, R.; Sathyanarayana, V.; Bullas, D.; Said, E.	2016	A dedicated PEG service can improve mortality and clinical outcome	Unable to obtain full text
Osaki, Y.; Morita, Y.; Miyamoto, Y.; Furuta, K.; Furuya, H.	2016	Pseudobulbar palsy as an early feature in movement disorders clinic	Insufficient Information about Condition
Prudlo, J.; Kasper, E.; Teipel, S.; Bvºttner, A.; Neumann, M.	2016	Rare forms of sporadic 4R-tauopathies with prominent motor neuron features easy to mistake for ALS-FTD	Unable to obtain full text
Shahgholi, L.; De Jesus, S.; Paterson, A.; Deeb, W.; McFarland, N.R.; Hegland, K.	2016	Subjective report does not predict objective swallow impairment in atypical Parkinsonian syndromes	Unable to obtain full text
Tilley, E.; McLoughlin, J.; Koblar, S. A.; Doeltgen, S. H.; Stern, C.; White, S.; Peters, M. D.	2016	Effectiveness of allied health therapy in the symptomatic management of progressive supranuclear palsy: a systematic review	Insufficient information on dysphagia presentation
Tripathi, S.M.; Singh, P.; Soni, R.; Tripathi, R.K.; Tiwari, S.C.	2016	A case of "rapidly Progressive" Progressive supranuclear palsy	Insufficient Information about Condition
Trufanov, Y.	2016	swallowing difficulties in patients with Parkinson's disease and Parkinsonian syndromes	Unable to obtain full text
Gomez-Caravaca, M.T.; Cáceres-Redondo, M.T.; Huertas-Fernández, I.; Vargas-González, L.; Carrillo, F.; Carballo, M.; Mir, P.	2015	The use of botulinum toxin in the treatment of sialorrhea in parkinsonian disorders	Wrong Outcomes
Lembeck, M.; Corcoran, A.M.	2015	Goals of care related to peg tubes and the role of sub-specialists in decision-making	Insufficient Information about Condition
Aerts, MB; Esselink, RAJ; Abdo, WF; Meijer, FJA; Drost, G; Norgren, N; Janssen, MJR; Borm, GF; Bloem, BR; Verbeek, MM	2015	Ancillary investigations to diagnose parkinsonism: a prospective clinical study	Insufficient Information about Condition
Respondek, G.; Kurz, C.; Stamelou, M.; Ferguson, L.W.; Rajput, A.; Chiu, W.Z.; Van Swieten, J.C.; Troakes, C.; Al Sarraj, S.; Gelpi, E.; Gaig, C.; Tolosa, E.; Wagenpfeil, S.; Giese, A.; Arzberger, T.; Höglinger, G.	2015	Benign progressive supranuclear palsy: A clinico-pathological analysis of cases with prolonged survival	Unable to obtain full text
Suttrup, I.; Suntrup, S.; Hamacher, C.; Oelenberg, S.; Siemer, M.-L.; Bauer, J.; Domagk, D.; Dziewas, R.; Warnecke, T.	2015	Prevalence and patterns of esophageal dysphagia in patients with parkinson's disease or atypical parkinsonism	Unable to obtain full text

Tilley, E.; White, S.; Peters, M.; Koblar, S.A.; Doeltgen, S.; McLoughlin, J.	2015	Effectiveness of allied health therapy in the symptomatic management of progressive supranuclear palsy: A systematic review	Unable to obtain full text
Tomita, S.; Oeda, T.; Umemura, A.; Kohsaka, M.; Park, K.; Yamamoto, K.; Sugiyama, H.; Mori, C.; Inoue, K.; Fujimura, H.; Sawada, H.	2015	Impact of Aspiration Pneumonia on the Clinical Course of Progressive Supranuclear Palsy: A Retrospective Cohort Study	Insufficient Information about Condition
Alonso-Canovas, A.; Lopez-Sendon, J.L.; Rabano, A.; Gomez Blazquez, E.; Garcia-Ribas, G.; Gerardo-Pian, H.; Garcia-Villanueva, M.; Garcia-Caldentey, J.; Garcia-Ruiz, zP.; Garcia De Yebenes Prous, J.; Martinez Castrillo, J.C.	2014	The two faces of atypical Parkinsonism diagnosis: Clinico-pathological cases	Wrong patient population
Moore, T.; Guttman, M.	2014	Challenges Faced by Patients With Progressive Supranuclear Palsy and their Families	Insufficient Information about Condition
Rodriguez-Leyva, I.; Calderon-Garciduev±as, A.; Santoyo, M.; Chi, E.; Medina-Mier, V.; Jimenez-Capdeville, M.	2014	Progressive supranuclear palsy (PSP): Diagnosis through skin biopsy	Unable to obtain full text
Varanese, S.; Di Ruscio, P.; Ben M' Barek, L.; Thomas, A.; Onofrj, M.	2014	Responsiveness of dysphagia to acute L-Dopa challenge in progressive supranuclear palsy	Wrong Study Design
Varanese, S.; Di Ruscio, P.; Thomas, A.; Onofrj, M.	2014	Levodopa response in four patients with progressive supranuclear palsy	Unable to obtain full text
Allinson, K.S.J.; Scotton, W.; Chaal, S.; Rowe, J.; O'Donovan, D.G.	2013	A choreiform movement disorder due to progressive supranuclear palsy	Unable to obtain full text
Becker, A.; Gleason, L.; Medina-Walpole, A.	2013	Progressive supranuclear palsy: A case of mistaken identity	Unable to obtain full text
Dash, S. K.	2013	Zolpidem in progressive supranuclear palsy	Insufficient Information about Condition
dell'Aquila, C.; Zoccolella, S.; Cardinali, V.; de Mari, M.; Iliceto, G.; Tartaglione, B.; Lamberti, P.; Logroscino, G.	2013	Predictors of survival in a series of clinically diagnosed progressive supranuclear palsy patients	Insufficient Information about Condition
Erro, R.; Barone, P.; Moccia, M.; Amboni, M.; Vitale, C.	2013	Abnormal eating behaviors in progressive supranuclear palsy	Insufficient Information about Condition
Furuya, H.; Watanabe, A.; Arahata, H.; Sasagasako, N.; Sakai, M.; Umemoto, G.; Kikuta, T.; Fujii, N.	2013	Long-term prognosis of swallowing difficulty observed in Parkinson's disease and related disorders	Unable to obtain full text
Golbe, Lawrence I.	2013	Diagnosis and management of progressive supranuclear palsy	Insufficient Information about Condition
Honig, L.; Honig, M.; Vonsattel, J.P.	2013	Incidental progressive supranuclear palsy neuropathology on autopsy	Insufficient Information about Condition
Iwasaki, Y.; Mori, K.; Ito, M.; Tatsumi, S.; Mimuro, M.; Yoshida, M.	2013	An autopsied case of progressive supranuclear palsy presenting with cerebellar ataxia and severe cerebellar involvement	Insufficient Information about Condition
Lücking, C.; Ceballos-Baumann, A.; Wagner-Sonntag, E.	2013	Unexpected epiglottical movements in patients with parkinsonian syndromes: Endoscopic findings and clinical implications	Unable to obtain full text
Kobayashi, Z.; Akaza, M.; Ishihara, S.; Tomimitsu, H.; Inadome, Y.; Arai, T.; Akiyama, H.; Shintani, S.	2013	Thalamic hypoperfusion in early stage of progressive supranuclear palsy (Richardson's syndrome): report of an autopsy-confirmed case	Insufficient Information about Condition
Komai, K.; Ishida, C.; Takahashi, K.; Motozaki, Y.; Ikeda, T.; Nozaki, I.; Tagami, A.	2013	Hypercapnic respiratory failure in patients with progressive supranuclear palsy	Insufficient Information about Condition
Nübling, G.; Lorenzl, S.	2013	Critical discussion of the use of percutaneous endocopic gastrotomiy in patients with progressive supranuclear palsy (PSP)	Unable to obtain full text
Nunomura, J.I.; Matsunaga, M.; Ono, H.	2013	Importance of idiopathic normal pressure hydrocephalus (iNPH) in the field of movement disorders	Insufficient Information about Condition
Saleem, T. Z.; Higginson, I. J.; Chaudhuri, K. R.; Martin, A.; Burman, R.; Leigh, P. N.	2013	Symptom prevalence, severity and palliative care needs assessment using the Palliative Outcome Scale: a cross-sectional study of patients with Parkinson's disease and related neurological conditions	Insufficient Information about Condition
Chen, S.; Juneja, S.; Jaffe, S.L.	2012	Combined zolpidem and carbidopa/levodopa treatment of progressive supranuclear palsy (PSP): A case report with video documentation	Unable to obtain full text
Jindal, N.; Shamkuwar, M. K.; Kaur, J.; Berry, S.	2012	Efficacy of ƒÄyurvedic treatment using Pa√±cakarma combined with balance exercises for disability and balance in progressive supranuclear palsy	Insufficient Information about Condition
Nagao, S.; Yokota, O.; Nanba, R.; Takata, H.; Haraguchi, T.; Ishizu, H.; Ikeda, C.; Takeda,	2012	Progressive supranuclear palsy presenting as primary lateral sclerosis but lacking parkinsonism, gaze palsy, aphasia, or dementia	Insufficient Information about Condition

N.; Oshima, E.; Sakane, K.; Terada, S.; Ihara, Y.; Uchitomi, Y.			
Nishida, N.; Hata, Y.; Ohtani, M.	2012	Progressive supranuclear palsy with unusual pathology	Unable to obtain full text
Noulas, N.; Karkala, E.; Maliamanis, D.; Panagopoulos, N.; Kouvalakidou, A.	2012	Anaesthesia management of a patient with steele-richardson-olszewski syndrome	Unable to obtain full text
Tomita, S.; Oeda, T.; Nakano, H.; Umemura, A.; Hayashi, R.; Yamamoto, K.; Sawada, H.	2012	Levodopa responsiveness for progressive supranuclear palsy can predict early onset of aspiration pneumonia	Insufficient Information about Condition
Velentzas, I.; Seferis, H.; Afentouli, P.; Torrens, M.; Tagaris, G.A73	2012	Two years observation after pedunculopontine nucleus (PPN) DBS surgery of two female patients with progressive supranuclear palsy (PSP)	Insufficient Information about Condition
Abhinav, K.; Marsh, L.; Crain, B.; Reich, S. G.; Biglan, K.	2011	Co-existence of Parkinson's disease and progressive supranuclear palsy: case report and a review of the literature	Wrong patient population
Stamelou, Maria; Rubio-Agusti, Ignacio; Quinn, Niall; Bhatia, Kailash	2011	Characteristic constant groaning in late stage progressive supranuclear palsy: A case report	Insufficient Information about Condition
Moore, T.F.; Guttman, M.	2011	PSP: A research study to improve support	Unable to obtain full text
Hensler, M.; Abright, C.; Nubling, G.; Lorenzl, S.	2011	Progressive supranuclear palsy and cortico-basal degeneration - Atypical parkinsonian disorders with impications for palliative medicine	Insufficient Information about Condition
Iwasaki, Y.; Mori, K.; Ito, M.; Mimuro, M.; Yoshida, M.	2011	[An autopsied case of progressive supranuclear palsy, initially diagnosed as spinocerebellar degeneration with severe olivopontocerebellar involvement]	Insufficient Information about Condition
Koyano, S.; Baba, Y.; Kuroiwa, Y.; Yagishita, S.	2011	An autopsy case of argyrophilic grains in progressive supranuclear palsy (PSP) lesion, clinically suspected normal pressure hydrocephalus (NPH) or PSP	Unable to obtain full text
Poppe, I.; De Vil, L.; Cras, P.	2011	Care needs in patients with PSP	Wrong study design
Suttrup, I.; Oelenberg, S.; Hamacher, C.; Dziewas, R.; Warnecke, T.	2011	Endoscopic L-Dopa-test for Parkinsonian dysphagia (a pilot study)	Insufficient Information about Condition
Toyoshima, Y.; Miyahara, H.; Yonemochi, Y.; Nakajima, T.; Kakita, A.; Takahashi, H.	2011	Two autopsy cases of progressive supranuclear palsy with severe pallido-nigro-luysial atrophy and less tau-pathology	Unable to obtain full text
Alfonsi, E.; Merlo, I. M.; Ponzio, M.; Montomoli, C.; Tassorelli, C.; Biancardi, C.; Lozza, A.; Martignoni, E.	2010	An electrophysiological approach to the diagnosis of neurogenic dysphagia: implications for botulinum toxin treatment	Wrong Study Design
Stenner, A.; Reichel, G.	2010	Botulinum toxin treatment of sialorrhea in 25 patients suffering from Parkinson's disease, MSA, PSP, and brain injury in early childhood	Unable to obtain full text
Iwase, T.; Mizuno, T.; Sato, S.; Ojika, K.; Mimuro, M.; Yoshida, M.; Hashizume, Y.	2010	A case of centenarian with progressive supranuclear palsy	Unable to obtain full text
Kouri, N.; Hassan, A.; Murray, M.E.; Dickson, D.W.	2010	Neuropathologic features of corticobasal degeneration presenting as corticobasal syndrome or Richardson's syndrome	Insufficient Information about Condition
Lorenzl, S.; Deutschenbaur, L.; Hensler, M.	2010	Effect of rasagiline in patients with progressive supranuclear palsy (PSP)	Unable to obtain full text
Saleem, T.Z.; Martin, A.; Leigh, P.N.; Higginson, I.J.	2010	Unknown group of movement disorders with palliative care needs-parkinson's plus syndromes: Multiple system atrophy (MSA) and progressive supranuclear palsy (PSP)	Unable to obtain full text
Shprecher, D.; Evans, R.	2010	Dysphagia as a presenting symptom of progressive supranuclear palsy	Unable to obtain full text
De Verdal, M.; Castelnovo, G.; Renard, D.; Labauge, P.	2009	Periaqueductal T2-weighted MRI hyperintensity in early stage progressive supranuclear palsy	Unable to obtain full text
Hardwick, A.; Rucker, J. C.; Cohen, M. L.; Friedland, R. P.; Gustaw-Rothenberg, K.; Riley, D. E.; Leigh, R. J.	2009	Evolution of oculomotor and clinical findings in autopsy-proven Richardson syndrome	Insufficient Information about Condition
Saleem, T.Z.; Leigh, P.N.; Martin, A.; Stewart, F.	2009	Assessment of palliative care needs in people affected by advanced Parkinson's disease and related disorders	Unable to obtain full text
Nijboer, H.; Dautzenberg, P. L. J.	2009	Progressieve supranucleaire verlamming Interventie middels acetylcholineesteraseremmer? = Progressive supranucleair palsy: Acetylcholineeserase - inhibitor a possible therapy	Insufficient Information about Condition
Stamelou, M.; Christ, H.; Oertel, W.H.; Höglinger, G.U.	2009	Hypodipsia discriminates PSP from PD and MSA	Unable to obtain full text

Troche, M. S.; Fernandez, H. H.; Okun, M. S.; Rodriguez, R. L.; Rosenbek, J. C.; Sapienza, C. M.	2009	Rehabilitation of dysphagia in progressive supranuclear palsy	Unable to obtain full text
Ahmed, Z.; Josephs, K. A.; Gonzalez, J.; DelleDonne, A.; Dickson, D. W.	2008	Clinical and neuropathologic features of progressive supranuclear palsy with severe pallido-nigro-luysial degeneration and axonal dystrophy	Insufficient Information about Condition
Kaphan, E.; Pellissier, J. F.; Rey, M.; Robert, D.; Auphan, M.; Ali Ch√©rif, A.	2008	[Esophageal achalasia, sleep disorders and chorea in a tauopathy without ophthalmoplegia, parkinsonian syndrome, nor dementia (progressive supranuclear palsy?): clinicopathological study]	Wrong Study Design
Karceski, S.	2008	Progressive supranuclear palsy	Wrong study design
O'Sullivan, S. S.; Massey, L. A.; Williams, D. R.; Silveira-Moriyama, L.; Kempster, P. A.; Holton, J. L.; Revesz, T.; Lees, A. J.	2008	Clinical outcomes of progressive supranuclear palsy and multiple system atrophy	Insufficient Information about Condition
Donker Kaat, L.; Boon, A. J.; Kamphorst, W.; Ravid, R.; Duivenvoorden, H. J.; van Swieten, J. C.	2007	Frontal presentation in progressive supranuclear palsy	Insufficient Information about Condition
Kovari, E.; Burkhardt, K.; Lobrinus, J. A.; Bouras, C.	2007	Lewy body dysphagia	Wrong patient population
Saeki, H.; Rino, Y.; Takanashi, Y.; Wada, N.; Yukawa, N.; Sasaki, K.; Arai, H.; Kanari, M.; Ino, H.; Fujii, K.; Imada, T.	2006	A case of intractable hemorrhage after self-removal of percutaneous endoscopic gastrostomy tube	Insufficient Information about Condition
Aiba, I.; Saito, Y.; Tamakoshi, A.; Matsuoka, Y.	2005	[Prognosis of patients with progressive supranuclear palsy]	Unable to obtain full text
Noda, K.; Kobayashi, T.; Matsuoka, S.; Takanashi, M.; Kanazawa, A.; Mizuno, Y.	2005	[A 65-year-old man with rigid-bradykinetic parkinsonism, vertical gaze palsy, difficulty of eye-lid opening, and marked pseudo-bulbar palsy]	Insufficient Information about Condition
Alvarez-Gonzalez, E.; Maragoto-Rizo, C.; Arteche-Prior, M.; Perez-Parra, S.; Carballo, M.; Alvarez-Gonzalez, L.	2004	A clinical and epidemiological description of a series of patients diagnosed as suffering from progressive supranuclear palsy	Unable to obtain full text
Diroma, C.; Dell'Aquila, C.; Fraddosio, A.; Lamberti, S.; Mastronardi, R.; Russo, I.; De Mari, M.; Iliceto, G.	2003	Natural history and clinical features of progressive supranuclear palsy: a clinical study	Insufficient Information about Condition
Goetz, C. G.	2003	[Progressive supranuclear palsy]	Insufficient Information about Condition
Goetz, C. G.; Leurgans, S.; Lang, A. E.; Litvan, I.	2003	Progression of gait, speech and swallowing deficits in progressive supranuclear palsy	Insufficient Information about Condition
Mochizuki, A.; Ueda, Y.; Komatsuzaki, Y.; Tsuchiya, K.; Arai, T.; Shoji, S.	2003	Progressive supranuclear palsy presenting with primary progressive aphasia--clinicopathological report of an autopsy case	Insufficient Information about Condition
Nath, U.; Ben-Shlomo, Y.; Thomson, R. G.; Lees, A. J.; Burn, D. J.	2003	Clinical features and natural history of progressive supranuclear palsy: a clinical cohort study	Insufficient Information about Condition
Nozaki, S.; Ichihara, N.; Yuasa, T.	2003	Management for dysphagia in patients with intractable neurological disorders: The current state and the ward of neurology in national hospitals	Insufficient Information about Condition
Schrag, A.; Selai, C.; Davis, J.; Lees, A. J.; Jahanshahi, M.; Quinn, N.	2003	Health-related quality of life in patients with progressive supranuclear palsy	Insufficient Information about Condition
Bloch, F.; Welter, M. L.; du Montcel, S. T.; Bonnet, A. M.; Bensimon, G.; Nnipps Study Grp	2002	Dysphagia, a major cause of death, is predictible in multiple system atrophy, not in progressive supranuclear palsy	Unable to obtain full text
Sjöström, A.; Holmberg, B.; Strang, P.	2002	Parkinson-plus patients -- an unknown group with severe symptoms	Insufficient Information about Condition
Fujino, Y.; Nakajima, M.; Tsuboi, Y.; Baba, Y.; Yamada, T.	2002	[Clinical effectiveness of tandospirone citrate (5-HT1A agonist) on patients with progressive supranuclear palsy]	Unable to obtain full text
Miyaoka, T.; Seno, H.; Inagaki, T.; Horiguchi, J.	2002	Fluvoxamine for the treatment of depression and parkinsonism in progressive supranuclear palsy	Insufficient Information about Condition
Muller, J.; Wenning, G. K.; Verny, M.; McKee, A.; Chaudhuri, K. R.; Jellinger, K.; Poewe, W.; Litvan, I.	2001	Progression of dysarthria and dysphagia in postmortem-confirmed Parkinsonian disorders	Insufficient Information about Condition
Ichihara, N.; Ichihara, S. I.; Fujii, S.; Touge, T.; Nishioka, M.	2000	[An assessment of dysphagia using videofluorography in Parkinson's disease and progressive supranuclear palsy]	Unable to obtain full text
Frattali, C. M.; Sonies, B. C.; Chi-Fishman, G.; Litvan, I.	1999	Effects of physostigmine on swallowing and oral motor functions in patients with progressive supranuclear palsy: A pilot study	Wrong Outcomes

Kulczycki, J.; Kuran, W.	1998	[Progressive supranuclear palsy, diagnostic problems in the light of own case]	Insufficient Information about Condition
Tamai, Sergio; Almeida, Osvaldo P.	1997	Nortriptyline for the treatment of depression in progressive supranuclear palsy	Unable to obtain full text
Leopold, N. A.; Kagel, M. C.	1997	Dysphagia in progressive supranuclear palsy: radiologic features	Wrong Study Design
Nakamura, T.; Yoritaka, A.; Sumino, S.; Suzuki, H.; Mori, H.; Suda, K.; Takubo, H.; Mizuno, Y.	1997	[A 70-year-old man with a progressive gait disturbance and gaze palsy]	Insufficient Information about Condition
Purcell, L.L.; Reich, S.G.	1997	Progressive supranuclear palsy	Insufficient Information about Condition
Yokoji, I.; Nakamura, S.; Ikeda, T.	1997	[A case of progressive supranuclear palsy associated with bilateral vocal cord abductor paralysis]	Wrong study design
Barclay, C. L.; Duff, J.; Sandor, P.; Lang, A. E.	1996	Limited usefulness of electroconvulsive therapy in progressive supranuclear palsy	Insufficient Information about Condition
De Bruin, V. S.; Machado, C.; Howard, R. S.; Hirsch, N. P.; Lees, A. J.	1996	Nocturnal and respiratory disturbances in Steele-Richardson-Olszewski syndrome (progressive supranuclear palsy)	Insufficient Information about Condition
Engel, P. A.	1996	Treatment of progressive supranuclear palsy with amitriptyline: therapeutic and toxic effects	Insufficient Information about Condition
Litvan, I.; Mangone, C. A.; McKee, A.; Verny, M.; Parsa, A.; Jellinger, K.; D'Olhaberriague, L.; Chaudhuri, K. R.; Pearce, R. K.	1996	Natural history of progressive supranuclear palsy (Steele-Richardson-Olszewski syndrome) and clinical predictors of survival: a clinicopathological study	Insufficient Information about Condition
Tetrud, J. W.; Golbe, L. I.; Forno, L. S.; Farmer, P. M.	1996	Autopsy-proven progressive supranuclear palsy in two siblings	Insufficient Information about Condition
Feldman, R.G.; McKee, A.C.	1993	A 75-year-old man with right-sided rigidity, dysarthria, and abnormal gait	Wrong patient population
Feldman, R. G.; McKee, A. C.	1993	Case records of the Massachusetts General Hospital. Weekly clinicopathological exercises. Case 46-1993. A 75-year-old man with right-sided rigidity, dysarthria, and abnormal gait	Duplicate
Hayashi, M.; Isozaki, E.; Suga, M.; Horiguchi, S.; Hayashida, T.	1992	[A case of progressive supranuclear palsy presenting mouth opening difficulty with tonic contraction of the orbicularis oris muscle]	Unable to obtain full text
Jankovic, J.; Wooten, M.; Van der Linden, C.; Jansson, B.	1992	Low body weight in Parkinson's disease	Insufficient Information about Condition
Brocker, P.; Vinsot, V.; Lagrange, M.; Falcot, J.; Chatelica, W.	1991	[Deglutition disorders and progressive supra nuclear paralysis. Value of subcutaneous administration of apomorphine]	Insufficient Information about Condition
Akashi, T.; Arima, K.; Maruyama, N.; Ando, S.; Inose, T.	1989	Severe cerebral atrophy in progressive supranuclear palsy: a case report	Unable to obtain full text
Campbelltaylor, I.; Tamas, I.	1988	DYSARTHRIA AND DYSPHAGIA IN THE EARLY STAGES OF PROGRESSIVE SUPRANUCLEAR PALSY	Unable to obtain full text
Golbe, L. I.; Davis, P. H.; Schoenberg, B. S.; Duvoisin, R. C.	1988	Prevalence and natural history of progressive supranuclear palsy	Insufficient Information about Condition
Izzo, K. L.; DiLorenzo, P.; Roth, A.	1986	Rehabilitation in progressive supranuclear palsy: case report	Insufficient Information about Condition
Trzepacz, Paula T.; Murcko, Anita C.; Gillepsie, M. Patrice	1985	Progressive supranuclear palsy misdiagnosed as schizophrenia	Insufficient Information about Condition
Calzetti, S.; Gemignani, F.; Lechi, A.; Pietrini, V.; Tagliavini, F.	1981	Progressive supranuclear palsy in the course of subclavian steal syndrome	Unable to obtain full text
Rafal, R. D.; Grimm, R. J.	1981	Progressive supranuclear palsy: functional analysis of the response to methysergide and antiparkinsonian agents	Unable to obtain full text
Trevisan, C.; Testa, G. F.; Angelini, C.	1975	[Progressive supranuclear palsy: report of two cases (author's transl)]	Unable to obtain full text
Ishino, H.; Ikeda, H.; Otsuki, S.	1974	Progressive supranuclear palsy--review of the literature with presentation of a case	Insufficient Information about Condition
Mano, Y.; Watanabe, H.; Nagata, Y.; Nishigaki, S.	1974	An autopsied case of progressive supranuclear palsy (Japanese)	Unable to obtain full text
Steele, J. C.	1970	Progressive supranuclear palsy. Report of a Thai patient	Unable to obtain full text
David, N. J.; Mackey, E. A.; Smith, J. L.	1968	Further observations in progressive supranuclear palsy	Unable to obtain full text
Anastasopoulos, G.; Routsonis, C.; Constas, C. G.	1967	[Oculo-facial-cervical dystonia (progressive supranuclear palsy)]	Unable to obtain full text
